# Supplementary material for: Protocol for a single-centre mixed-method pre–post single-arm feasibility trial of a culturally appropriate 6-week pulmonary rehabilitation programme among adults with functionally limiting chronic respiratory diseases in Malawi
Source: BMJ Open. 2022 Jan 31;12(1):e057538. doi: 10.1136/bmjopen-2021-057538 (PMC8804676; doi:10.1136/bmjopen-2021-057538)
Supplement: Supplementary data [file bmjopen-2021-057538supp001.pdf]

Supplementary file 1: Participant Information Sheet (English version)

1. **Study title**

Feasibility and acceptability of pulmonary rehabilitation for adults with functionally limiting chronic respiratory disease in Malawi: A mixed-methods single-arm interventional prospective cohort study

2. **Invitation**

I would like to invite you to take part in a research study. Before you decide you need to understand why the research is being done and what it would involve for you. Please take time to read the following information carefully. Ask questions if anything you read is not clear or would like more information. Take time to decide whether or not to take part.

3. **Purpose of study**

The study aims to (1) establish the acceptability and feasibility of an intervention in the management of adults with functionally limiting chronic respiratory diseases (CRDs) in Malawi, called pulmonary rehabilitation (PR), and (2) to design a locally appropriate PR program for adults with functionally limiting CRDs in Malawi. A similar study has recently been conducted in Uganda.

4. **Why have I been invited?**

You have been invited to participate in this study because you have a chronic respiratory disease.

5. **Do I have to take part?**

Participation in the study is entirely voluntary. It is up to you to decide. We will describe the study and go through the information sheet, which we will give to you. We will then ask you to sign a consent form to show you agreed to participate. You are free to withdraw at any time, without giving a reason and this will not affect the standard of care you receive for your disease.

6. **What will happen to me if I take part?**

You will be invited to participate and complete a pulmonary rehabilitation (PR) programme run at Queen Elizabeth Central Hospital (QECH) by a team of healthcare professionals including physiotherapists, nurses and doctors. Before the programme begins, you will be assessed for your eligibility to participate in the study and to establish your baseline characteristics. The programme will run for 6 weeks, 2 days of a supervised session each week at QECH and 1 day of unsupervised session as your home, 2 hours each session. The programme will involve exercises such as walking, education such as knowledge about your disease, and behaviour change such as smoking cessation. The exercises will be tailored and progressed according to each participant's abilities. Other 9 participants of a similar condition like yours will also participate in the study together with you. After the 6 weeks of the programme, you will be assessed again for any changes that might have happened due to the programme. We may also assess you during the programme. The assessments may involve, among others, your lung function, your exercise capacity/tolerance, your quality of life, and vital signs such as blood pressure and pulse rate. There will be social interaction with other participants during the programme. There will also be rest periods and provision of light food and soft drinks as refreshments. We will try to

make the programme as much interesting and enjoyable as possible to everyone. At times, we will collect information from you about the programme through audio-recorded interviews and group discussions. All data collected will be managed with confidentiality by the study team.

7. Will there be any risks involved in the study?

PR is safe except under certain circumstances, such as if you have uncontrolled heart disease or exacerbations of your respiratory diseases. The assessment we will conduct prior to your participation in the program will help us establish the programme's safety to you. In the case where exacerbations happen to you during the programme, we will immediately stop the programme and refer you to the medical personnel for immediate treatment. Since the programme will happen within the premises if the hospital, such referral process will be easier for you. The programme will not stop you from continuing your normal standard of care including taking your medications. Otherwise, with all safety measures in place, we don't expect any risks during the study.

8. Will there be any benefits involved in being in the study?

We cannot promise the study will help you but we know that PR has well-established benefits. We, therefore, hope that it will lead to better outcomes of your disease and health.

9. What will happen to the findings of the study?

We will record the information we collect from you using a computer. This information will be transferred to a computer database with your identifiers removed, so that you cannot be identified from this information. The information will be stored securely for 5-years.

The results of the study will be shared at research meetings (in Malawi and overseas) and will be published in medical journals, so that other healthcare professionals and patients can benefit from our findings. You will not be identified in such avenues too.

10. Who is organizing the study?

The principal investigator of the study is Mr. Fanuel Bickton, a trained physiotherapist and research intern working at the Malawi-Liverpool-Wellcome Trust Clinical Research Programme (MLW). He and the study are being supervised by Dr. Jamie Rylance from MLW and Mr. Enock Chisati from the University of Malawi.

11. Expenses/payments

You will be compensated for your travel expenses (MWK1500.00 per study visit) to participate in the study.

12. Source of funding for the study

The study is funded by the Malawi-Liverpool-Wellcome Trust Clinical Research Programme through the Lung Health research group.

13. What happens if I change my mind?

If you agree to join the study you can change your mind and withdraw your consent at any time. If you have any questions about this study, please contact any of the following individuals:

Principle investigator:

Mr. Fanuel Bickton:
Cell: +265 982 55 23 53
E-mail: fmbickton@stud.medcol.mw

Supervisors:

1. Dr. Jamie Rylance:
   Cell: +265 (0) 996 80 46 11
   E-mail: jamie.rylance@lstmed.ac.uk

2. Mr. Enock Chisati:
   Cell: +265 (0) 888 16 82 84
   E-mail: echisati@medcol.mw / echisati@gmail.com

The local ethics committee (COMREC) has reviewed and approved this study – any problems that cannot be addressed by the study team, should be directed to:

COMREC Secretariat, College of Medicine, P/Bag 360, Chichiri, Blantyre 3, Malawi; Tel: +265 1871911 ext. 334; E-mail: comrec@medcol.mw

You will be given a copy of this sheet to keep, together with a copy of your consent form.
