# Supplementary material for: Protocol for a single-centre mixed-method pre–post single-arm feasibility trial of a culturally appropriate 6-week pulmonary rehabilitation programme among adults with functionally limiting chronic respiratory diseases in Malawi
Source: BMJ Open. 2022 Jan 31;12(1):e057538. doi: 10.1136/bmjopen-2021-057538 (PMC8804676; doi:10.1136/bmjopen-2021-057538)
Supplement: Supplementary data [file bmjopen-2021-057538supp002.pdf]

Supplementary file 2: Topic Guide for Pre-PR In-Depth Interviews (English version)

The guide may be adapted during the research in response to emerging issues.
Overall structure of the interview:

i. Consent of the participant to take part in this trial
ii. **Recorder started**
iii. Self-introduction by the researcher
iv. Explanation to the participant that they should treat the information they hear as confidential
v. Detailed explanation of what PR is and what it involves (see definition and explanation below)
vi. Structured interview around participant's barriers and enablers for participating and completing a PR programme
vii. **Recorder stopped**

PR summary (to use for quick reference for key elements of PR for either interviewer or participant where necessary):

Pulmonary rehabilitation is the use of exercise, education, and behaviour change to improve health and quality of life in people with chronic respiratory diseases such as COPD, asthma and p-TBLD. The exercise component may include walking, the education component may include knowledge about nutrition and patient's disease and how patients can manage the symptoms themselves, and behaviour change may include adopting and adhering to health-enhancing behaviours such as smoking cessation. PR begins and ends with a thorough assessment of the patient. PR is recommended in international guidelines for the management of chronic respiratory diseases; it has been found to improve the patient's quality of life by, among others: reducing shortness of breath, increasing exercise tolerance, promoting a sense of well-being, and decreasing the number of hospitalizations. A typical PR programme runs for at least six weeks and involves weekly two supervised sessions at the hospital and one unsupervised session at the patient's home. Each session takes about 2 hours. Participants interact with each other during the sessions. There are also rest periods and refreshments in form of light food and soft drinks are provided during these periods. We try to make the sessions as much interesting and enjoyable as possible to everyone.

1. **Participant's experience with breathing difficulties**
   1.1 How can you describe your breathing problem?
   1.2 How has your life changed because of breathing difficulties? What is it in your daily life worries you about your breathing?
   1.3 What do you do to relieve your breathing problem?

2. **Participant's views on enabling and limiting factors to undertaking a PR program:** 2.1 What factors do you think would enable you attend and complete the programme?
   2.2 What factors do you think would prevent you from attending and completing the programme?
3. **Participant's attitudes and views on different elements/components of PR:**
   3.1 The programme will be taking place at the hospital's gym, either in the morning or afternoon of the selected 2 days. What can you say and suggest about the location, days, and timing of the programme?

3.2 PR involves group work. What do you think about this?

3.3 One feature of pulmonary rehabilitation is a regular, tailored exercise session. What do you think about this?

3.4 Another feature of the programme is education which may include the importance of smoking cessation along with support to do so. What can you say about this?

3.5 Participating in a PR programme would mean you travelling from your home to a PR centre. What do you say about this?

2.9 A participant group attending a PR program may be a mixture of men and women. How do you feel about that?

2.10 Professionals delivering a PR program can be male or female. What do you say about that?

2.11 Due to the nature of the PR program, you may have to do it in public sometimes. How do you feel about that?

4. Conclusion:

4.1 If you accepted to participate in a PR program, which aspects of your health or life would you most like to see improvement in?

4.2 What are your hopes and doubts about the PR program?

4.3 How can we make the programme successful or attainable for you?

4.4 Do you have additional comments?
