# Supplementary material for: Protocol for a single-centre mixed-method pre–post single-arm feasibility trial of a culturally appropriate 6-week pulmonary rehabilitation programme among adults with functionally limiting chronic respiratory diseases in Malawi
Source: BMJ Open. 2022 Jan 31;12(1):e057538. doi: 10.1136/bmjopen-2021-057538 (PMC8804676; doi:10.1136/bmjopen-2021-057538)
Supplement: Supplementary data [file bmjopen-2021-057538supp003.pdf]

Supplementary file 3: Topic guide for post-PR in-depth Interviews (English version)

The guide may be adapted during the research in response to emerging issues.
Overall structure of the interview:

i. Consent of the participant to take part in this trial
ii. **Recorder started**
iii. Self-introduction by the researcher
iv. Explanation to the participant that they should treat the information they hear as confidential
v. Detailed introduction of the interview (see below)
vi. Structured interview around the impact of PR on patient's disease and/or quality of life, barriers, and enablers for participating and completing the trial's PR programme, the willingness/motivation to undertake the programme again in the future, the willingness to continue the programme at home, and the willingness to recommend the programme to other patients with similar condition
vii. **Recorder stopped**

Introduction:

Thank you for participating and completing a PR programme in the trial that has just ended. Now, this part of the trial aims to establish your experiences with the programme including its impact on your health or quality of life, your views on what went well and what went wrong with the programme, your recommendations on how to improve the programme, and your motivation/willingness to undertake the programme again in the future and to continue it at your home. Please, note that there aren't any right or wrong answers. I am just trying to gather your opinions and experiences. I am asking the same questions to your friends with whom you undertook the programme (if IDIs are used) and the information you share with me is confidential.

1. Impact of the PR program on participants

1.1 How are you feeling now?

1.2 How does you disease affect you since you have taken part in the rehabilitation programme?

1.3 What has changed since last time I spoke to you?

1.4 Which aspects of your activities have changed?

1.5 How do you now feel about taking part in activities such as going out/walking, etc.?

1.6 How do you feel about your breathlessness now?

2. Recommendations

2.1 What do you think went well and what did not go well with the programme?

2.2 What recommendations can you suggest improving the program?

2.3 In the future, for you to attend and complete a PR programme, what circumstances would have to change for you?

3. Maintenance of PR program

3.1 Would you consider continuing the programme at your home?

4. Conclusion

4.1 Are there any other issues you would like to raise?
