# Supplementary material for: Protocol for a single-centre mixed-method pre–post single-arm feasibility trial of a culturally appropriate 6-week pulmonary rehabilitation programme among adults with functionally limiting chronic respiratory diseases in Malawi
Source: BMJ Open. 2022 Jan 31;12(1):e057538. doi: 10.1136/bmjopen-2021-057538 (PMC8804676; doi:10.1136/bmjopen-2021-057538)
Supplement: Author's manuscript [file bmjopen-2021-057538.draft_revisions.pdf]

BMJ Open is committed to open peer review. As part of this commitment we make the peer review history of every article we publish publicly available.

When an article is published we post the peer reviewers' comments and the authors' responses online. We also post the versions of the paper that were used during peer review. These are the versions that the peer review comments apply to.

The versions of the paper that follow are the versions that were submitted during the peer review process. They are not the versions of record or the final published versions. They should not be cited or distributed as the published version of this manuscript.

BMJ Open is an open access journal and the full, final, typeset and author-corrected version of record of the manuscript is available on our site with no access controls, subscription charges or pay-per-view fees (http://bmjopen.bmj.com).

If you have any questions on BMJ Open's open peer review process please email info.bmjopen@bmj.com

BMJ Open

Protocol for a single-centre mixed-methods pre-post single-arm feasibility trial of a culturally appropriate six-week pulmonary rehabilitation programme among adults with functionally limiting chronic respiratory diseases in Malawi

Journal:	*BMJ Open*
Manuscript ID	bmjopen-2021-057538
Article Type:	Protocol
Date Submitted by the Author:	20-Sep-2021

Complete List of Authors:	Bickton, Fanuel; Malawi-Liverpool-Wellcome Trust Clinical Research Programme, Lung Health Research Group<br>Mankhokwe, Talumba ; Queen Elizabeth Central Hospital, Physiotherapy Department<br>Nightingale, Rebecca; Liverpool School of Tropical Medicine, Clinical Sciences; Malawi Liverpool Wellcome Trust Programme, Lung Health Group<br>Fombe, Cashon ; Queen Elizabeth Central Hospital, Physiotherapy Department<br>Mitengo, Martha ; Queen Elizabeth Central Hospital, Physiotherapy Department<br>Mwahimba, Langsfield ; Queen Elizabeth Central Hospital, Physiotherapy Department<br>Lipita, Wilfred ; Queen Elizabeth Central Hospital, Physiotherapy Department<br>Wilde, Laura; University of Leicester, Department of Respiratory Sciences; NIHR Leicester Biomedical Research Centre Respiratory Diseases, Centre for Exercise and Rehabilitation Science<br>Pina, Ilaria ; University of Leicester, Department of Respiratory Sciences; NIHR Leicester Biomedical Research Centre Respiratory Diseases, Centre for Exercise and Rehabilitation Science<br>Yusuf, Zainab; University of Leicester, Department of Respiratory Sciences; University Hospitals of Leicester NHS Trust, Centre for Exercise and Rehabilitation Sciences<br>Ahmed, Zahira ; University of Leicester, Department of Respiratory Sciences; NIHR Leicester Biomedical Research Centre Respiratory Diseases, Centre for Exercise and Rehabilitation Science<br>Kamponda, Martin ; Queen Elizabeth Central Hospital, Medicine Department<br>Limbani, Felix ; Malawi-Liverpool-Wellcome Trust Clinical Research Programme<br>Shannon, Harriet; University College London Institute of Child Health, Physiotherapy, RCCA, III<br>Chisati, Enock ; University of Malawi, Physiotherapy<br>Barton, Andy; University of Leicester, Department of Respiratory Sciences<br>Free, Robert; University of Leicester, Department of Respiratory Sciences<br>Steiner, michael ; Institute for Lung Health, NIHR Leicester Biomedical

	Research Centre – Respiratory, University of Leicester, Glenfield Hospital,<br>Matheson, Jesse; University of Sheffield, Department of Economics<br>Manise, Adrian ; University of Leicester, Department of Respiratory Sciences<br>Singh, Sally; University Hospitals of Leicester NHS Trust, Cardiac/Pulmonary Rehabilitation<br>Rylance, Jamie; Liverpool School of Tropical Medicine,<br>Orme, Mark; University of Leicester, Respiratory Sciences

Keywords:	Rehabilitation medicine < INTERNAL MEDICINE, Chronic airways disease < THORACIC MEDICINE, Respiratory physiology < THORACIC MEDICINE

SCHOLARONE™
Manuscripts

Protocol for a single-centre mixed-methods pre-post single-arm feasibility trial of a culturally appropriate six-week pulmonary rehabilitation programme among adults with functionally limiting chronic respiratory diseases in Malawi

Fanuel M Bickton[1,2,3], Talumba Mankhokwe[4], Rebecca Nightingale[2], Cashon Fombe[4], Martha Mitengo[4], Langsfield Mwahimba[4], Wilfred Lipita[4], Laura J Wilde[5,6], Ilaria Pina[5,6], Zainab Yusuf[5,6], Zahira Ahmed[5,6], Martin Kamponda[4], Felix Limbani[1], Harriet Shannon[3], Enock Chisati[7], Andy Barton[5], Rob C Free[5], Michael C Steiner[5,6], Jesse Matheson[8], Adrian Manise[9], Sally J Singh[5,6], Jamie Rylance[1,2*], Mark W Orme[5,6*]

1. Malawi-Liverpool-Wellcome Trust Clinical Research Programme, Blantyre, Malawi
2. Liverpool School of Tropical Medicine, Liverpool, United Kingdom
3. UCL GOS Institute of Child Health, London, United Kingdom
4. Queen Elizabeth Central Hospital, Blantyre, Malawi
5. Department of Respiratory Sciences, University of Leicester, Leicester, United Kingdom
6. Centre for Exercise and Rehabilitation Science, NIHR Leicester Biomedical Research Centre-Respiratory, Leicester, United Kingdom
7. Kamuzu University of Health Sciences, Blantyre, Malawi
8. Department of Economics, University of Sheffield, Sheffield, United Kingdom
9. NIHR Leicester Biomedical Research Centre, University Hospitals of Leicester NHS Trust, Leicester, United Kingdom

*Joint senior author

Correspondence to Fanuel M Bickton (Email: fbickton@mlw.mw; Twitter: @fmbickton)

Word count: 2 593

For peer review only - http://bmjopen.bmj.com/site/about/guidelines.xhtml

Abstract

Introduction: Malawi has a substantial burden of chronic respiratory diseases (CRDs) which cause significant morbidity and loss of economic productivity, affecting patients, families, and health systems alike. Pulmonary rehabilitation (PR) is a highly recommended non-pharmacological intervention in the clinical management of people with CRDs. However, Malawi lacks published evidence on the implementation of PR for people with CRDs. This trial will test the feasibility and acceptability of a culturally appropriate hospital-based PR programme among adults with functionally limiting CRDs at Queen Elizabeth Central Hospital in Blantyre, Malawi.

Methods and analysis: This is a single-centre mixed-methods pre-post single-arm feasibility trial. Twenty-five patients aged ≥18 years, with a spirometry confirmed diagnosis of a CRD and breathlessness of ≥2 on the modified Medical Research Council dyspnoea scale, will be consecutively recruited. Their baseline lung function, exercise tolerance, and health status will be assessed; including spirometry, incremental shuttle walk test and chronic obstructive pulmonary disease assessment test, respectively. Pre-trial semi-structured in-depth interviews will explore their experiences of living with CRD and potential enablers and barriers to their PR uptake. Along with international PR guidelines, these data will inform culturally appropriate delivery of PR. We initially propose a six-week, twice-weekly, supervised centre-based PR programme, with an additional weekly home-based non-supervised session. Following programme completion (after six weeks), repeat assessments of lung function, exercise tolerance and health status will be conducted. Quantitative changes in clinical outcomes will be described in relation to published minimal clinically important differences. Post-trial semi-structured interviews will capture participants' perceived impact of the PR programme on their quality of life, enablers, and barriers to fully engaging with the programme, and allow iteration of its design.

For peer review only - http://bmjopen.bmj.com/site/about/guidelines.xhtml

Ethics and dissemination: Ethical approval for this trial was obtained from University of Malawi College of Medicine Research and Ethics Committee (COMREC), Blantyre, Malawi (protocol number: P.07/19/2752), and University of Leicester Research Ethics Committee, Leicester, United Kingdom (ethics reference: 31574). The results of the trial will be disseminated through oral presentations at local and international scientific conferences or seminars and publication in a peer-reviewed journal. We will also engage the participants who complete the PR trial and the Science Communication Department at Malawi-Liverpool-Wellcome Trust Clinical Research Programme to organise community outreach activities within Blantyre to educate communities about CRDs and PR. We will also broadcast our trial results through national radio station programmes such as the weekly "*Thanzi la Onse*" (Health of All) programme by Times Radio Malawi. We will formally present our trial results to Blantyre District Health Office and Malawi Ministry of Health.

Trial registration number: ISRCTN13836793

Strengths and limitations of this trial:

- The trial will use participants' experiences to help inform the design of a culturally appropriate pulmonary rehabilitation programme.
- Acceptability of such services will be key to reducing the morbidity associated with functionally limiting chronic respiratory disease.
- This is a single-centre mixed-methods pre-post single-arm feasibility trial. As such, it will not generate definitive data on efficacy, but guide future evaluation in Malawi and potentially other low-income and middle-income countries, including likely sample size requirements.

For peer review only - http://bmjopen.bmj.com/site/about/guidelines.xhtml

Introduction

Low- and middle-income countries (LMICs) bear a disproportionately high burden of the global morbidity and mortality caused by chronic respiratory diseases (CRDs), including asthma, chronic obstructive pulmonary disease (COPD), bronchiectasis, and post-tuberculosis lung disease (p-TBLD) (1). Known risk factors include tobacco smoking, outdoor air pollution, household smoke exposure, occupational dust exposure, and pulmonary tuberculosis (2). Significant chronic morbidity and loss of economic productivity are related to CRDs, which burden patients, families and health systems (3).

A recent meta-analysis of Malawi data identified a high burden of chronic respiratory symptoms and abnormal spirometry data (particularly low forced vital capacity) in both children and adults (4). Notably, even successful tuberculosis (TB) treatment is still frequently followed by long-term cough and shortness of breath, leading to impaired quality of life, and placing a continued and sustained financial burden upon patients and their families (5, 6). Unfortunately, while drugs are the mainstay of treatment, they do not reduce the excess decline in lung function that is common in CRDs (7). Poor availability and affordability of drugs for CRDs in LMICs limits their impact, and effective non-pharmacological treatments could help mitigate this deficit (8).

The World Health Organization (WHO) recognized the global burden of non-communicable diseases (NCDs) through its “Rehabilitation 2030: A Call for Action” initiative (9). Rehabilitation is a set of interventions to address limitations in everyday physical, mental, and social functioning due to ageing, or a specific health condition such as chronic disease or injury (10). For individuals with CRDs, pulmonary rehabilitation (PR) is one of the core treatment modalities. This is a programme of exercise training, education, and behaviour management, designed to improve the physical and psychological condition of people with CRDs and to promote the long-term adherence of health-enhancing behaviours (11). Notably, in people with COPD, PR is supported by high-quality evidence of improvement in symptoms (dyspnoea, fatigue, anxiety, depression), exercise tolerance, and overall health-

For peer review only - http://bmjopen.bmj.com/site/about/guidelines.xhtml

related quality of life (12). There is also evidence supporting PR for other CRDs including asthma (13), p-TBLD (14) and bronchiectasis (15). In high-income countries, PR significantly reduces the direct costs of COPD by decreasing healthcare system usage, particularly unplanned hospital admissions (16). In LMICs, low-cost modifications can reduce the costs of specialist equipment, potentially allowing wider access and increased feasibility (17, 18).

Current PR evidence is predominantly based on studies from high-income countries (19). Clinical PR services are not widely available in LMICs (20), where significant modifications may be required due to differences in resources, awareness, culture, healthcare configuration, and target disease epidemiology (21). A systematic review of PR in sub-Saharan Africa demonstrated few trials, and generally low-quality evidence for efficacy, and no published data from Malawi (3). Our proposed trial aims to investigate the feasibility and acceptability of PR in Malawi, with the following specific objectives:

1. To co-design, with service users and stakeholders, a locally appropriate PR programme for adult patients with functionally limiting CRDs in Malawi.
2. To examine participants' recruitment, retention, engagement, adverse events, drop-outs, loss to follow up, and acceptability. This trial adapts the definition of "acceptability" by Sekhon et al (22); the extent to which participants in this trial consider the PR programme to be appropriate, based on their experienced cognitive and emotional responses to the intervention. Elements of "acceptability" will include the participants' attitudes, burden, perceived effectiveness, ethicality, intervention coherence, opportunity costs, and self-efficacy (22).
3. To describe changes in participants' lung function, exercise capacity and health status after their completion of the PR programme.

Findings of this trial will inform the design of a multi-centre randomized controlled trial of PR in Malawi to help address the broder question, "In Africa (and LMICs, more broadly), what is the feasibility and effectiveness of PR?"

For peer review only - http://bmjopen.bmj.com/site/about/guidelines.xhtml

Methods and analysis

The trial will be conducted, analysed and reported according to the Standard Protocol Items: Recommendations for Interventional Trials (SPIRIT) statement (23). The trial is prospectively registered on the ISRCTN website (https://doi.org/10.1186/ISRCTN13836793).

Trial design

The proposed feasibility trial will use a single-centre mixed-methods pre-post single-arm design and will be conducted in three phases as follows:

- Phase 1: pre-trial qualitative work to inform the modifications required to make PR specific to the Malawi context
- Phase 2: trial of the six-week PR programme
- Phase 3: post-trial quantitative and qualitative evaluation to determine the feasibility and acceptability of PR among participants, followed by identification of future modifications to the PR programme by the PR delivery team.

Trial place

The trial will be conducted at a single site, namely Queen Elizabeth Central Hospital (QECH), Blantyre, Malawi.

Trial period

Phase 1 is expected to run from October 2021 to November 2021. Phase 2 is expected to run from November 2021 to February 2022. Phase 3 is expected to run from February 2022 to March 2022.

Target population, sample size, recruitment, and eligibility

The trial population will comprise patients presenting with functionally limiting CRDs such as p-TBLD, COPD, asthma, and bronchiectasis, at QECH. Twenty-five participants will be recruited in both phase 1 and phase 2, with phase 2 participants being either some or all of phase 1 participants or completely different ones (depending on patients' consent to participate in both phases), divided into groups of five per PR class or session. This sample size was chosen

For peer review only

pragmatically based on sample sizes of previous similar feasibility and acceptability studies of PR in Africa (24, 25) and United Kingdom (26), ranging from 7 to 29 participants. Participants will be consecutively sampled and recruited from the chest clinic and medical wards at QECH. Inclusion criteria are: aged 18 years or older; a spirometry confirmed diagnosis of CRD; functional limitation due to breathlessness reaching a score of ≥2 on the modified Medical Research Council (mMRC) dyspnoea scale (27). Exclusion criteria are those who do not meet the inclusion criteria and any of the following: have significant cardiovascular, neurological, orthopaedic, cognitive, or any other condition that would compromise participation in the rehabilitation programme; have an active infection including TB and coronavirus disease 2019 (COVID-19); have respiratory disease which is thought primarily to originate from COVID-19; are unable to provide informed consent.

Data collection

Phase 1

Face-to-face semi-structured in-depth interviews with eligible participants will explore their experiences of living with CRD, including functional limitations, and identify potential enablers and barriers to participating in or adhering to PR. These interviews will be conducted in a quiet, private room. Interviews are expected to last for up to one hour and will be audio-recorded with prior consent. A topic guide (Supplementary material 1) will direct the content of the interview and will include participants' experiences with breathing difficulties, anticipated enabling and limiting factors to undertaking a PR programme, attitudes and views on different elements or components of the programme, and suggestions for making the programme culturally acceptable to them.

Phase 2

As already mentioned, phase 2 will recruit either some or all of phase 1 participants or completely different participants, depending on patients' consent to participate in both phases. A case report form will collect the following data from phase 2 participants:

- **Demographic variables** including age, sex, ethnicity, education and employment status;

For peer review only - http://bmjopen.bmj.com/site/about/guidelines.xhtml

- **Lung health** variables consisting of smoking status (pack years), biomass fuel exposure, primary respiratory diagnosis, time since diagnosis in years, number of CRD exacerbations and hospitalisations within the last 12 months, spirometric measurements (pre- and post-bronchodilator FEV_1, FVC, and FEV_1/FVC ratio); comorbidities and treatments;
- **Anthropometric measurements** of height (using a Holtain stadiometer), weight (using calibrated weight scale), and body mass index (BMI);
- **Health status** variables including participants' perceived respiratory disability due to dyspnoea (using the mMRC dyspnoea scale (28)), health-related quality of life (using the COPD Assessment Test (29)), subjective experience of fatigue (using the Checklist Individual Strength fatigue subscale (30)), and psychological wellbeing (using the Hospital Anxiety and Depression Scale (31));
- **Physical fitness** variables including exercise tolerance (using the incremental shuttle walk test (32)), with before- and after-test measurements of oxygen saturation by pulse oximetry (SpO_2), blood pressure (by a sphygmomanometer), Borg rating of breathlessness (33), heart rate (by a heart rate monitor), and Borg rate of perceived exertion (33). We will also assess lower extremity muscular strength (using five-repetition sit-to-stand test (34)) and hamstring flexibility (using the chair sit-and-reach test (35)).

Interview data from phase 1 will be used to culturally adapt the PR programme. We initially propose a six-week, twice-weekly, supervised centre-based PR programme, with an additional weekly home-based non-supervised session. The programme will be delivered in a group format and will consist of about an hour of exercise training and another hour of health education. The PR sessions or classes will be run in the physiotherapy department at QECH. Participants will be provided with refreshments and reimbursed for transport, time and inconvenience at rates mandated by the local research ethics committee. The intensity of the exercise regime will be tailored to each participant's exercise capacity (i.e., based on each participant's performance on the incremental shuttle walk test) (36), with a gradual increase according to each patient's improvement over the course of PR.

For peer review only

At each session, participants will complete a set of exercises designed to improve aerobic fitness, muscle strength, endurance, and flexibility. Established principles of exercise training will be used during exercise prescription (37). Each participant will also be given an information sheet containing illustrations and instructions written in Chichewa (local language) for their weekly home-based exercise session. Participants will be requested to keep a standardized exercise and physical activity diary at home. The PR programme will not interfere with the participants' routine medical care from the primary care and/or referring teams.

The education component will be delivered to participants and their families or caregivers, which address: patients as active participants in their health care; understanding of the physical and psychological changes that occur with chronic illness, and coping strategies for these (38). Education will be delivered either in group or individual sessions depending on the sensitivity of the topic and insights from phase 1. Table 1 below lists some of the topics that may be covered (39).

Table 1: Education topics

**Topics that may be suitable for group sessions**	**Topics that may require individual consultation**
Information about the lungs, lung diseases and respiratory medications	Smoking cessation
Exacerbations: prevention and management including action plans	Inhaler technique
Benefits of physical activity and exercise	Nutrition
Causes and management of breathlessness	Oxygen therapy
Coping with chronic lung disease, management of depression, anxiety, and panic attacks	Instruction in airway clearance techniques
Communication with health professionals	Issues related to travelling with lung disease
Community resources including home care options	Sexual intimacy
	Continence
	Advanced care planning and end-of-life decision-making

For peer review only

For peer review only - http://bmjopen.bmj.com/site/about/guidelines.xhtml

Phase 3

Repeat assessments of participants' lung function, anthropometry (weight, height, body mass index), submaximal exercise tolerance, and health status will be conducted. Post-trial interviews with participants will also be conducted using a topic guide (Supplementary material 2), to assess the acceptability of the programme to participants. Participants will also be asked for what went well and what might be improved in the programme. All participants who complete the six-week PR programme will be invited to participate in these interviews. The interviews are expected to last for up to one hour and will be audio-recorded with prior consent.

Patient and public involvement

As described in phase 1 and 2 sections, patients will be involved in the design of a culturally appropriate PR programme for this trial using their data collected through in-depth interviews prior to PR trial phase. After the trial, we will recruit a patient and public involvement (PPI) representative (patient expert) from the trial participants' group who can help, in a formal role, with the dissemination of trial findings and PPI in future studies. Currently, the protocol has received both internal and external expert input.

Data management

Quantitative data will be entered into a database using Research Electronic Data Capture (REDCap), a web-based platform (40) with database access controlled via a secure password-protected interface. All participants will be allocated a trial-specific identification code. Data entered on the REDCap database will be subject to real-time data entry validation and electronic checks led by the Independent Data Monitoring Committee, established at the University of Leicester, United Kingdom.

It is anticipated that the interviews will be conducted in Chichewa (most used local language) in which case the interview audio files will be translated and transcribed verbatim into English. Data will be anonymised at the time of translation and transcription. Personal data including names, locations and dates will be removed. All anonymised data will be stored on a password-

For peer review only

For peer review only - http://bmjopen.bmj.com/site/about/guidelines.xhtml

encrypted computer at Malawi-Liverpool-Wellcome Trust and stored securely for at least five years after which it will be permanently deleted.

Quantitative data analysis and presentation

Quantitative data will be imported to IBM SPSS Statistics 27 for descriptive analysis. The average pre-post PR outcome differences or changes in clinical outcomes will be represented as relative and absolute changes. Non-parametric statistical testing (Wilcoxon signed-rank test (41)) will be used to explore differences, with the caveat that this feasibility trial is not designed a priori for statistical power to detect differences or changes in clinical outcomes. In addition, as the trial lacks a control group with which to compare any recorded difference in clinical outcomes, we will be unable to attribute any pre-post PR difference or change in clinical outcomes to PR. However, where appropriate, the average changes in clinical outcomes will be discussed in relation to published minimal clinically important differences (42). Results from quantitative data analysis will be presented in tables and/or graphs, with mean (standard deviation), median (inter-quartile range) or frequency (percentage) used to report summary data.

Qualitative data analysis and presentation

Anonymised interview transcripts will be imported into QSR NVivo software platform for thematic analysis (43). The analysis will be performed collaboratively between researchers to encourage a breadth and depth of analysis from different perspectives and viewpoints. Initially, the researchers will collaborate to develop a preliminary coding framework. Data coding will be performed by the researchers separately, using an iterative inductive approach (43) across all qualitative data. In summary, the researchers will broadly follow the steps outlined by Braun and Clarke (43), including going through a process of familiarization with the data by reading and re-reading transcripts while making reflective notes on the literal content, looking closely at words used by participants, interpreting what the data meant by assigning initial codes or classifications to segments of text, and exploring relationships between these classifications and developing

For peer review only - http://bmjopen.bmj.com/site/about/guidelines.xhtml

core themes. Results from qualitative data analysis will be presented in narrative form based on key themes established during the analysis, supplemented by participants' quotations.

Adverse events

For the purposes of this work, an adverse event is defined as any unfavourable or unintended sign or response associated with participating in the PR programme of this trial. Due to anticipated thorough participant assessment (including risk assessment) and individualised nature of PR in this trial, adverse events are not anticipated. However, if such events do occur, the programme will be stopped immediately for the affected participant so that they can be given appropriate management, which may include referral to the medical team. All adverse events will be registered on the adverse event log and CRF. Serious adverse events will be reported to the Clinical Research Support Unit (CRSU) at Malawi-Liverpool-Wellcome Trust Clinical Research Programme, COMREC, and University of Leicester Research Ethics Committee, for guidance where required.

Ethics and dissemination of the results

Ethical approval for this trial was obtained from University of Malawi College of Medicine Research and Ethics Committee (COMREC), Blantyre, Malawi (protocol number: P.07/19/2752), and University of Leicester Research Ethics Committee, Leicester, United Kingdom (ethics reference: 31574). The results of the trial will be disseminated through oral presentations at local and international scientific conferences or seminars and publication in a peer-reviewed journal. We will also engage the participants who complete the PR trial and the Science Communication Department at Malawi-Liverpool-Wellcome Trust Clinical Research Programme to organise community outreach activities within Blantyre to educate communities about CRDs and PR. We will also broadcast our trial results through national radio station programmes such as the weekly "Thanzi la Onse" (Health of All) programme by Times Radio Malawi. We will formally present our trial results to Blantyre District Health Office and Malawi Ministry of Health.

For peer review only - http://bmjopen.bmj.com/site/about/guidelines.xhtml

For peer review only

Authors' contributions

FMB, JR, EC, MWO, SJS and HS conceived the trial. All authors (FMB, JR, EC, TM, RN, MWO, CF, MM, LM, WL, LW, IP, ZY, ZA, MK, FL, HS, AB, RCF, MCS, JM, AM, SJS) contributed to the design of the trial. FMB drafted the manuscript which all authors revised critically for important intellectual content. All authors approved the final version to be published and are accountable for all aspects of the work.

Funding

This work was supported by the National Institute for Health Research (NIHR) (using the UK's Official Development Assistance (ODA) Funding) and Wellcome [221465/Z/20/Z] under the NIHR-Wellcome Partnership for Global Health Research; the Royal Society of Tropical Medicine and Hygiene (RSTMH) 2019 Small Grants programme (funded by NIHR; award/grant number N/A); the Academy of Medical Sciences Global Challenges Research Fund (GCRF) Networking Grant Scheme (GCRFNGR5\1242); and the NIHR- Global Health Research Group on Respiratory Rehabilitation (Global RECHARGE) (17/63/20) using UK aid from the UK Government to support global health research.

For peer review only - http://bmjopen.bmj.com/site/about/guidelines.xhtml

Competing interests

The authors declare no competing interests.

References

1. Meghji J, Mortimer K, Agusti A, Allwood BW, Asher I, Bateman ED, et al. Improving lung health in low-income and middle-income countries: from challenges to solutions. Lancet. 2021;397(10277):928-40.
2. van Kampen SC, Wanner A, Edwards M, Harries AD, Kirenga BJ, Chakaya J, et al. International research and guidelines on post-tuberculosis chronic lung disorders: a systematic scoping review. BMJ Glob Health. 2018;3(4):e000745.
3. Bickton FM, Fombe C, Chisati E, Rylance J. Evidence for pulmonary rehabilitation in chronic respiratory diseases in sub-Saharan Africa: a systematic review. Int J Tuberc Lung Dis. 2020;24(10):991-9.
4. Nightingale R. Non-communicable respiratory disease in Malawi: a systematic review and meta-analysis. Malawi Medical Journal. 2020;32:64-73.
5. Meghji J, Lesosky M, Joekes E, Banda P, Rylance J, Gordon S, et al. Patient outcomes associated with post-tuberculosis lung damage in Malawi: a prospective cohort study. Thorax. 2020;75(3):269.
6. Meghji J, Gregorius S, Madan J, Chitimbe F, Thomson R, Rylance J, et al. The long term effect of pulmonary tuberculosis on income and employment in a low income, urban setting. Thorax. 2021;76(4):387.
7. Montuschi P, Malerba M, Santini G, Miravitlles M. Pharmacological treatment of chronic obstructive pulmonary disease: from evidence-based medicine to phenotyping. Drug Discov Today. 2014;19(12):1928-35.
8. Kibirige D, Sanya RE, Nantanda R, Worodria W, Kirenga B. Availability and affordability of medicines and diagnostic tests recommended for management of asthma

For peer review only

and chronic obstructive pulmonary disease in sub-Saharan Africa: a systematic review. Allergy Asthma Clin Immunol. 2019;15:14.
9. Gimigliano F, Negrini S. The World Health Organization "Rehabilitation 2030: a call for action". Eur J Phys Rehabil Med. 2017;53(2):155-68.
10. Cieza A, Causey K, Kamenov K, Hanson SW, Chatterji S, Vos T. Global estimates of the need for rehabilitation based on the Global Burden of Disease study 2019: a systematic analysis for the Global Burden of Disease Study 2019. Lancet. 2021;396(10267):2006-17.
11. Spruit MA, Singh SJ, Garvey C, ZuWallack R, Nici L, Rochester C, et al. An official American Thoracic Society/European Respiratory Society statement: key concepts and advances in pulmonary rehabilitation. Am J Respir Crit Care Med. 2013;188(8):e13-64.
12. Bolton CE, Bevan-Smith EF, Blakey JD, Crowe P, Elkin SL, Garrod R, et al. British Thoracic Society guideline on pulmonary rehabilitation in adults. Thorax. 2013;68 Suppl 2:ii1-30.
13. Majd S, Apps L, Chantrell S, Hudson N, Eglington E, Hargadon B, et al. A Feasibility Study of a Randomized Controlled Trial of Asthma-Tailored Pulmonary Rehabilitation Compared with Usual Care in Adults with Severe Asthma. The Journal of Allergy and Clinical Immunology: In Practice. 2020;8(10):3418-27.
14. Ando M, Mori A, Esaki H, Shiraki T, Uemura H, Okazawa M, et al. The effect of pulmonary rehabilitation in patients with post-tuberculosis lung disorder. Chest. 2003;123(6):1988-95.
15. Patel S, Cole AD, Nolan CM, Barker RE, Jones SE, Kon S, et al. Pulmonary rehabilitation in bronchiectasis: a propensity-matched study. Eur Respir J. 2019;53(1).
16. Johnston K, Grimmer-Somers K. Pulmonary rehabilitation: overwhelming evidence but lost in translation? Physiother Can. 2010;62(4):368-73.
17. Griffiths TL, Phillips CJ, Davies S, Burr ML, Campbell IA. Cost effectiveness of an outpatient multidisciplinary pulmonary rehabilitation programme. Thorax. 2001;56(10):779-84.
18. Patel S, Palmer MD, Nolan CM, Barker RE, Walsh JA, Wynne SC, et al. Supervised pulmonary rehabilitation using minimal or specialist exercise equipment in COPD: a propensity-matched analysis. Thorax. 2021;76(3):264-71.
19. Habib GMM, Rabinovich R, Divgi K, Ahmed S, Saha SK, Singh S, et al. Systematic review of clinical effectiveness, components, and delivery of pulmonary rehabilitation in low-resource settings. NPJ Prim Care Respir Med. 2020;30(1):52.
20. Singh SJ, Halpin DMG, Salvi S, Kirenga BJ, Mortimer K. Exercise and pulmonary rehabilitation for people with chronic lung disease in LMICs: challenges and opportunities. Lancet Respir Med. 2019;7(12):1002-4.
21. Habib GM, Uzzaman MN, Malik P, Rabinovich R, Uddin A, Alam SR, et al. Engaging with stakeholders in a research programme to promote implementation of pulmonary rehabilitation in Bangladesh: Challenges and opportunities. J Glob Health. 2020;10(2):020384.
22. Sekhon M, Cartwright M, Francis JJ. Acceptability of healthcare interventions: an overview of reviews and development of a theoretical framework. BMC Health Services Research. 2017;17(1):88.

For peer review only

23. Chan AW, Tetzlaff JM, Altman DG, Laupacis A, Gøtzsche PC, Krleža-Jerić K, et al. SPIRIT 2013 statement: defining standard protocol items for clinical trials. Ann Intern Med. 2013;158(3):200-7.
24. Jones R, Kirenga BJ, Katagira W, Singh SJ, Pooler J, Okwera A, et al. A pre-post intervention study of pulmonary rehabilitation for adults with post-tuberculosis lung disease in Uganda. International journal of chronic obstructive pulmonary disease. 2017;12:3533-9.
25. Clarke H, Voss M. The role of a multidisciplinary student team in the community management of chronic obstructive pulmonary disease. Primary Health Care Research & Development. 2016;17(4):415-20.
26. Moore L, Hogg L, White P. Acceptability and feasibility of pulmonary rehabilitation for COPD: a community qualitative study. Primary Care Respiratory Journal. 2012;21(4):419-24.
27. NICE. Chronic obstructive pulmonary disease in over 16s: diagnosis and management. 2018.
28. Munari AB, Gulart AA, dos Santos K, Venâncio RS, Karloh M, Mayer AF. Modified Medical Research Council Dyspnea Scale in GOLD Classification Better Reflects Physical Activities of Daily Living. Respiratory Care. 2018;63(1):77.
29. Jones PW, Harding G, Berry P, Wiklund I, Chen WH, Kline Leidy N. Development and first validation of the COPD Assessment Test. Eur Respir J. 2009;34(3):648-54.
30. Vercoulen JH, Swanink CM, Fennis JF, Galama JM, van der Meer JW, Bleijenberg G. Dimensional assessment of chronic fatigue syndrome. J Psychosom Res. 1994;38(5):383-92.
31. Zigmond AS, Snaith RP. The hospital anxiety and depression scale. Acta Psychiatr Scand. 1983;67(6):361-70.
32. Singh SJ, Morgan MD, Scott S, Walters D, Hardman AE. Development of a shuttle walking test of disability in patients with chronic airways obstruction. Thorax. 1992;47(12):1019-24.
33. Mahler DA, Horowitz MB. Perception of breathlessness during exercise in patients with respiratory disease. Med Sci Sports Exerc. 1994;26(9):1078-81.
34. Jones SE, Kon SSC, Canavan JL, Patel MS, Clark AL, Nolan CM, et al. The five-repetition sit-to-stand test as a functional outcome measure in COPD. Thorax. 2013;68(11):1015.
35. Jones CJ, Rikli RE, Max J, Noffal G. The reliability and validity of a chair sit-and-reach test as a measure of hamstring flexibility in older adults. Res Q Exerc Sport. 1998;69(4):338-43.
36. Armstrong M, Vogiatzis I. Personalized exercise training in chronic lung diseases. Respirology. 2019;24(9):854-62.
37. Garvey C, Bayles MP, Hamm LF, Hill K, Holland A, Limberg TM, et al. Pulmonary Rehabilitation Exercise Prescription in Chronic Obstructive Pulmonary Disease: Review of Selected Guidelines: AN OFFICIAL STATEMENT FROM THE AMERICAN ASSOCIATION OF CARDIOVASCULAR AND PULMONARY REHABILITATION. J Cardiopulm Rehabil Prev. 2016;36(2):75-83.
38. Australia" LF. Pulmonary rehabilitation toolkit – importance of education. 2016
39. Jenkins S, Hill K, Cecins NM. State of the art: how to set up a pulmonary rehabilitation program. Respirology. 2010;15(8):1157-73.

For peer review only

For peer review only - http://bmjopen.bmj.com/site/about/guidelines.xhtml

40. Harris PA, Taylor R, Minor BL, Elliott V, Fernandez M, O'Neal L, et al. The REDCap consortium: Building an international community of software platform partners. J Biomed Inform. 2019;95:103208.
41. King AP, Eckersley RJ. Chapter 6 - Inferential Statistics III: Nonparametric Hypothesis Testing. In: King AP, Eckersley RJ, editors. Statistics for Biomedical Engineers and Scientists: Academic Press; 2019. p. 119-45.
42. McGlothlin AE, Lewis RJ. Minimal Clinically Important Difference: Defining What Really Matters to Patients. JAMA. 2014;312(13):1342-3.
43. Braun V, Clarke V. Using thematic analysis in psychology. Qualitative Research in Psychology. 2006;3(2):77-101.
44. Ritchie J SL. Qualitative Data Analysis for Applied Policy Research. In: Bryman, A. and Burgess, R., Eds., Anal. Qual. Data, Routledge, London, 173-194.
. 1994.

For peer review only

For peer review only - http://bmjopen.bmj.com/site/about/guidelines.xhtml

Supplementary material 1: Topic Guide for Pre-PR In-Depth Interviews

The guide may be adapted during the research in response to emerging issues.

Overall structure of the interview:

i. Consent of the participant to take part in this trial
ii. **Recorder started**
iii. Self-introduction by the researcher
iv. Explanation to the participant that they should treat the information they hear as confidential
v. Detailed explanation of what PR is and what it involves (see definition and explanation below)
vi. Structured interview around participant's barriers and enablers for participating and completing a PR programme
vii. **Recorder stopped**

About PR (read out verbatim):

Pulmonary rehabilitation is the use of exercise, education, and behaviour change to improve health and quality of life in people with chronic respiratory diseases such as COPD, asthma and p-TBLD. The exercise component may include walking, the education component may include knowledge about nutrition and patient's disease and how patients can manage the symptoms themselves, and behaviour change may include adopting and adhering to health-enhancing behaviours such as smoking cessation. PR begins and ends with a thorough assessment of the patient. PR is recommended in international guidelines for the management of chronic respiratory diseases; it has been found to improve the patient's quality of life by, among others: reducing shortness of breath, increasing exercise tolerance, promoting a sense of well-being, and decreasing the number of hospitalizations. A typical PR programme runs for at least six weeks and involves weekly two supervised sessions at the hospital and one unsupervised session at the patient's home. Each session takes about 2 hours. Participants interact with each other during the sessions. There are also rest periods and refreshments in form of light food and soft drinks are provided during these periods. We try to make the sessions as much interesting and enjoyable as possible to everyone.

1. **Participant's experience with breathing difficulties**
   1.1 How can you describe your breathing problem?
   1.2 How has your life changed because of breathing difficulties? What is it in your daily life worries you about your breathing?
   1.3 What do you do to relieve your breathing problem?

2. **Participant's views on enabling and limiting factors to undertaking a PR program:**
   2.1 What factors do you think would enable you attend and complete the programme?
   2.2 What factors do you think would prevent you from attending and completing the programme?
3. **Participant's attitudes and views on different elements/components of PR:**
   3.1 The programme will be taking place at the hospital's gym, either in the morning or afternoon of the selected 2 days. What can you say and suggest about the location, days, and timing of the programme?

For peer review only

3.2 PR involves group work. What do you think about this?
3.3 One feature of pulmonary rehabilitation is a regular, tailored exercise session. What do you think about this?
3.4 Another feature of the programme is education which may include the importance of smoking cessation along with support to do so. What can you say about this?
3.5 Participating in a PR programme would mean you travelling from your home to a PR centre. What do you say about this?
2.9 A participant group attending a PR program may be a mixture of men and women. How do you feel about that?
2.10 Professionals delivering a PR program can be male or female. What do you say about that?
2.11 Due to the nature of the PR program, you may have to do it in public sometimes. How do you feel about that?

4. Conclusion:

4.1 If you accepted to participate in a PR program, which aspects of your health or life would you most like to see improvement in?
4.2 What are your hopes and doubts about the PR program?
4.3 How can we make the programme successful or attainable for you?
4.4 Do you have additional comments?

For peer review only

Supplementary material 2: Topic guide for post-PR in-depth Interviews

The guide may be adapted during the research in response to emerging issues.

Overall structure of the interview:

i. Consent of the participant to take part in this trial
ii. **Recorder started**
iii. Self-introduction by the researcher
iv. Explanation to the participant that they should treat the information they hear as confidential
v. Detailed introduction of the interview (see below)
vi. Structured interview around the impact of PR on patient's disease and/or quality of life, barriers, and enablers for participating and completing the trial's PR programme, the willingness/motivation to undertake the programme again in the future, the willingness to continue the programme at home, and the willingness to recommend the programme to other patients with similar condition
vii. **Recorder stopped**

Introduction:

Thank you for participating and completing a PR programme in the trial that has just ended. Now, this part of the trial aims to establish your experiences with the programme including its impact on your health or quality of life, your views on what went well and what went wrong with the programme, your recommendations on how to improve the programme, and your motivation/willingness to undertake the programme again in the future and to continue it at your home. Please, note that there aren't any right or wrong answers. I am just trying to gather your opinions and experiences. I am asking the same questions to your friends with whom you undertook the programme (if IDIs are used) and the information you share with me is confidential.

1. **Impact of the PR program on participants:**

1.1 How are you feeling now?
1.2 How does you disease affect you since you have taken part in the rehabilitation programme?
1.3 What has changed since last time I spoke to you?
1.4 Which aspects of your activities have changed?
1.5 How do you now feel about taking part in activities such as going out/walking, etc.?
1.6 How do you feel about your breathlessness now?

2. **Recommendations**

2.1 What do you think went well and what did not go well with the programme?
2.2 What recommendations can you suggest improving the program?
2.3 In the future, for you to attend and complete a PR programme, what circumstances would have to change for you?

3. **Maintenance of PR program**

3.1 Would you consider continuing the programme at your home?

4. **Conclusion**

4.1 Are there any other issues you would like to raise?

For peer review only

BMJ Open

Protocol for a single-centre mixed-methods pre-post single-arm feasibility trial of a culturally appropriate six-week pulmonary rehabilitation programme among adults with functionally limiting chronic respiratory diseases in Malawi

Journal:	*BMJ Open*
Manuscript ID	bmjopen-2021-057538.R1
Article Type:	Protocol
Date Submitted by the Author:	02-Dec-2021

Complete List of Authors:	Bickton, Fanuel; Malawi-Liverpool-Wellcome Trust Clinical Research Programme, Lung Health Research Group<br>Mankhokwe, Talumba ; Queen Elizabeth Central Hospital, Physiotherapy Department<br>Nightingale, Rebecca; Liverpool School of Tropical Medicine, Clinical Sciences; Malawi Liverpool Wellcome Trust Programme, Lung Health Group<br>Fombe, Cashon ; Queen Elizabeth Central Hospital, Physiotherapy Department<br>Mitengo, Martha ; Queen Elizabeth Central Hospital, Physiotherapy Department<br>Mwahimba, Langsfield ; Queen Elizabeth Central Hospital, Physiotherapy Department<br>Lipita, Wilfred ; Queen Elizabeth Central Hospital, Physiotherapy Department<br>Wilde, Laura; University of Leicester, Department of Respiratory Sciences; NIHR Leicester Biomedical Research Centre Respiratory Diseases, Centre for Exercise and Rehabilitation Science<br>Pina, Ilaria ; University of Leicester, Department of Respiratory Sciences; NIHR Leicester Biomedical Research Centre Respiratory Diseases, Centre for Exercise and Rehabilitation Science<br>Yusuf, Zainab; University of Leicester, Department of Respiratory Sciences; University Hospitals of Leicester NHS Trust, Centre for Exercise and Rehabilitation Sciences<br>Ahmed, Zahira ; University of Leicester, Department of Respiratory Sciences; NIHR Leicester Biomedical Research Centre Respiratory Diseases, Centre for Exercise and Rehabilitation Science<br>Kamponda, Martin ; Queen Elizabeth Central Hospital, Medicine Department<br>Limbani, Felix ; Malawi-Liverpool-Wellcome Trust Clinical Research Programme<br>Shannon, Harriet; University College London Institute of Child Health, Physiotherapy, RCCA, III<br>Chisati, Enock ; University of Malawi, Physiotherapy<br>Barton, Andy; University of Leicester, Department of Respiratory Sciences<br>Free, Robert; University of Leicester, Department of Respiratory Sciences<br>Steiner, Michael; Institute for Lung Health, NIHR Leicester Biomedical

	Research Centre – Respiratory, University of Leicester, Glenfield Hospital,<br>Matheson, Jesse; University of Sheffield, Department of Economics<br>Manise, Adrian ; University of Leicester, Department of Respiratory Sciences<br>Singh, Sally; University Hospitals of Leicester NHS Trust, Cardiac/Pulmonary Rehabilitation<br>Rylance, Jamie; Liverpool School of Tropical Medicine,<br>Orme, Mark; University of Leicester, Respiratory Sciences
**Primary Subject Heading**:	Rehabilitation medicine
Secondary Subject Heading:	Rehabilitation medicine
Keywords:	Rehabilitation medicine < INTERNAL MEDICINE, Chronic airways disease < THORACIC MEDICINE, Respiratory physiology < THORACIC MEDICINE

SCHOLARONE™
Manuscripts

Protocol for a single-centre mixed-methods pre-post single-arm feasibility trial of a culturally appropriate six-week pulmonary rehabilitation programme among adults with functionally limiting chronic respiratory diseases in Malawi

Fanuel M Bickton[1,2,3], Talumba Mankhokwe[4], Rebecca Nightingale[2], Cashon Fombe[4], Martha Mitengo[4], Langsfield Mwahimba[4], Wilfred Lipita[4], Laura J Wilde[5,6], Ilaria Pina[5,6], Zainab K Yusuf[5,6], Zahira Ahmed[5,6], Martin Kamponda[4], Felix Limbani[1], Harriet Shannon[3], Enock Chisati[7], Andy Barton[5], Rob C Free[5], Michael C Steiner[5,6], Jesse Matheson[8], Adrian Manise[9], Sally J Singh[5,6], Jamie Rylance[1,2*], Mark W Orme[5,6*]

1. Malawi-Liverpool-Wellcome Trust Clinical Research Programme, Blantyre, Malawi
2. Liverpool School of Tropical Medicine, Liverpool, United Kingdom
3. UCL GOS Institute of Child Health, London, United Kingdom
4. Queen Elizabeth Central Hospital, Blantyre, Malawi
5. Department of Respiratory Sciences, University of Leicester, Leicester, United Kingdom
6. Centre for Exercise and Rehabilitation Science, NIHR Leicester Biomedical Research Centre-Respiratory, Leicester, United Kingdom
7. Kamuzu University of Health Sciences, Blantyre, Malawi
8. Department of Economics, University of Sheffield, Sheffield, United Kingdom
9. NIHR Leicester Biomedical Research Centre, University Hospitals of Leicester NHS Trust, Leicester, United Kingdom

*Joint senior author

Correspondence to Fanuel M Bickton (Email: fbickton@mlw.mw; Twitter: @fmbickton)

Word count: 2 968

For peer review only - http://bmjopen.bmj.com/site/about/guidelines.xhtml

Abstract

Introduction: Malawi has a substantial burden of chronic respiratory diseases (CRDs) which cause significant morbidity and loss of economic productivity, affecting patients, families, and health systems. Pulmonary rehabilitation (PR) is a highly recommended non-pharmacological intervention in the clinical management of people with CRDs. However, Malawi lacks published evidence on the implementation of PR for people with CRDs. This trial will test the feasibility and acceptability of a culturally appropriate hospital-based PR programme among adults with functionally limiting CRDs at Queen Elizabeth Central Hospital in Blantyre, Malawi.

Methods and analysis: This is a single-centre mixed-methods pre-post single-arm feasibility trial. Ten patients aged ≥18 years, with a spirometry confirmed diagnosis of a CRD and breathlessness of ≥2 on the modified Medical Research Council dyspnoea scale, will be consecutively recruited. Their baseline lung function, exercise tolerance, and health status will be assessed; including spirometry, incremental shuttle walk test and chronic obstructive pulmonary disease assessment test, respectively. Pre-trial semi-structured in-depth interviews will explore their experiences of living with CRD and potential enablers and barriers to their PR uptake. Along with international PR guidelines, these data will inform culturally appropriate delivery of PR. We initially propose a six-week, twice-weekly, supervised centre-based PR programme, with an additional weekly home-based non-supervised session. Using combination of researcher observation, interaction with the participants, field notes and informal interviews with the participants, we will assess the feasibility of running the programme in the following areas: participants' recruitment, retention, engagement, and protocol adherence. Following programme completion (after six weeks), repeat assessments of lung function, exercise tolerance and health status will be conducted. Quantitative changes in clinical outcomes will be described in relation to published minimal clinically important differences. Post-trial semi-structured interviews will capture participants' perceived impact of the PR programme on their quality of life,

For peer review only - http://bmjopen.bmj.com/site/about/guidelines.xhtml

enablers, and barriers to fully engaging with the programme, and allow iteration of its design.

Ethics and dissemination: Ethical approval for this trial was obtained from University of Malawi College of Medicine Research and Ethics Committee (COMREC), Blantyre, Malawi (protocol number: P.07/19/2752), and University of Leicester Research Ethics Committee, Leicester, United Kingdom (ethics reference: 31574). The results of the trial will be disseminated through oral presentations at local and international scientific conferences or seminars and publication in a peer-reviewed journal. We will also engage the participants who complete the PR trial and the Science Communication Department at Malawi-Liverpool-Wellcome Trust Clinical Research Programme to organise community outreach activities within Blantyre to educate communities about CRDs and PR. We will also broadcast our trial results through national radio station programmes such as the weekly "*Thanzi la Onse*" (Health of All) programme by Times Radio Malawi. We will formally present our trial results to Blantyre District Health Office and Malawi Ministry of Health.

Trial registration number: ISRCTN13836793

Strengths and limitations of this trial:

- Pre-trial interviews with participants will result in a "culturally adapted" intervention
- Post-trial interviews will result in a refined and more acceptable intervention
- The design of this trial will inform the design of a randomised controlled trial
- Small sample size and single-centredness will limit the external validity of this trial
- The cohort design of this trial will not ascertain a cause-and-effect relationship

For peer review only - http://bmjopen.bmj.com/site/about/guidelines.xhtml

Introduction

Low- and middle-income countries (LMICs) bear a disproportionately high burden of the global morbidity and mortality caused by chronic respiratory diseases (CRDs), including asthma, chronic obstructive pulmonary disease (COPD), bronchiectasis, and post-tuberculosis lung disease (p-TBLD) (1). Known risk factors include tobacco smoking, outdoor air pollution, household smoke exposure, occupational dust exposure, and pulmonary tuberculosis (2). Significant chronic morbidity and loss of economic productivity are related to CRDs, which burden patients, families and health systems (3).

A recent meta-analysis of Malawi data identified a high burden of chronic respiratory symptoms and abnormal spirometry data (particularly low forced vital capacity) in both children and adults (4). Notably, even successful tuberculosis (TB) treatment is still frequently followed by long-term cough and shortness of breath, leading to impaired quality of life, and placing a continued and sustained financial burden upon patients and their families (5, 6). Unfortunately, while drugs are the mainstay of treatment, they do not reduce the excess decline in lung function that is common in CRDs (7). Poor availability and affordability of drugs for CRDs in LMICs limits their impact, and effective non-pharmacological treatments could help mitigate this deficit (8).

The World Health Organization (WHO) recognized the global burden of non-communicable diseases (NCDs) through its “Rehabilitation 2030: A Call for Action” initiative (9). Rehabilitation is a set of interventions to address limitations in everyday physical, mental, and social functioning due to ageing, or a specific health condition such as chronic disease or injury (10). For individuals with CRDs, pulmonary rehabilitation (PR) is one of the core treatment modalities. This is a programme of exercise training, education, and behaviour management, designed to improve the physical and psychological condition of people with CRDs and to promote the long-term adherence of health-enhancing behaviours (11). Notably, in people with COPD, PR is supported by high-quality evidence of improvement in symptoms (dyspnoea, fatigue, anxiety, depression), exercise tolerance, and overall health-

For peer review only

For peer review only - http://bmjopen.bmj.com/site/about/guidelines.xhtml

related quality of life (12). There is also evidence supporting PR for other CRDs including asthma (13), p-TBLD (14) and bronchiectasis (15). In high-income countries, PR significantly reduces the direct costs of COPD by decreasing healthcare system usage, particularly unplanned hospital admissions (16). In LMICs, low-cost modifications can reduce the costs of specialist equipment, potentially allowing wider access and increased feasibility (17, 18).

Current PR evidence is predominantly based on studies from high-income countries (19). Clinical PR services are not widely available in LMICs (20), where significant modifications may be required due to differences in resources, awareness, culture, healthcare configuration, and target disease epidemiology (21). A systematic review of PR in southern Africa demonstrated few trials, and generally low-quality evidence for efficacy, and no published data from Malawi (3). Our proposed trial aims to investigate the feasibility and acceptability of PR in Malawi, with the following specific objectives:

1. To co-design, with service users and stakeholders, a locally appropriate PR programme for adult patients with functionally limiting CRDs in Malawi.
2. To examine feasibility (i.e., participants' recruitment, retention, engagement, protocol adherence) and acceptability of the programme. This trial adapts the definition of "acceptability" by Sekhon et al (22); the extent to which participants in this trial consider the PR programme to be appropriate, based on their experienced cognitive and emotional responses to the intervention. Elements of "acceptability" will include the participants' attitudes, burden, perceived effectiveness, ethicality, intervention coherence, opportunity costs, and self-efficacy (22).
3. To describe changes in participants' lung function, exercise capacity and health status after their completion of the PR programme.

Findings of this trial will inform the design of a multi-centre randomized controlled trial of PR in Malawi to help address the broader question, "In low-resource settings (i.e., like Malawi, settings with suboptimal healthcare service delivery for people with CRDs, underdeveloped healthcare infrastructure including lack of PR specialist equipment, paucity

For peer review only - http://bmjopen.bmj.com/site/about/guidelines.xhtml

of PR knowledge and expertise, and shortage of human resources for health including rehabilitation professionals)(23), what is the feasibility and effectiveness of PR?"

Methods and analysis

The trial will be conducted, analysed and reported according to the Standard Protocol Items: Recommendations for Interventional Trials (SPIRIT) statement (24). The trial is prospectively registered on the ISRCTN website (https://doi.org/10.1186/ISRCTN13836793).

Trial design

The proposed feasibility trial will use a single-centre mixed-methods pre-post single-arm design and will be conducted in three phases as follows:

- Phase 1: pre-trial qualitative work to inform the modifications required to make PR specific to the Malawi context
- Phase 2: trial of the six-week PR programme
- Phase 3: post-trial quantitative and qualitative evaluation to determine the feasibility and acceptability of PR among participants, followed by identification of future modifications to the PR programme by the PR delivery team.

Trial place

The trial will be conducted at a single site, namely Queen Elizabeth Central Hospital (QECH), Blantyre, Malawi.

Trial period

Phase 1 is expected to run from October 2021 to November 2021. Phase 2 is expected to run from November 2021 to February 2022. Phase 3 is expected to run from February 2022 to March 2022.

Target population, sample size, recruitment, and eligibility

The trial population will comprise patients presenting with functionally limiting CRDs such as p-TBLD, COPD, asthma, and bronchiectasis, at QECH. Ten participants will be recruited, and these

For peer review only - http://bmjopen.bmj.com/site/about/guidelines.xhtml

will be invited to participate in both phase 1 and phase 2 of the study. In phase 2 (the trial phase), they will be divided into groups of five per PR class or session. This sample size was chosen pragmatically based on previous PR studies of similar sample sizes (25, 26)(i.e., 7 and 12 participants, resepcetively). Participants will be consecutively sampled and recruited from the chest clinic and medical wards at QECH. Inclusion criteria are: aged 18 years or older; a spirometry confirmed diagnosis of any CRD such as COPD, post-tuberculosis lung disease, asthma and bronchiectasis; functional limitation due to breathlessness reaching a score of ≥2 on the modified Medical Research Council (mMRC) dyspnoea scale (27). Exclusion criteria are those who do not meet the inclusion criteria above; have acute or unstable conditions and medical complications (e.g., acute myocardial infarction, uncontrolled asthma, syncope, thrombosis, pulmonary oedema, uncontrolled arrhythmias causing symptoms or haemodynamic compromise, acute respiratory failure, mental impairment leading to inability to cooperate, etc) or any other condition that would compromise participation in the rehabilitation programme; have an active infection including TB and coronavirus disease 2019 (COVID-19); have respiratory disease which is thought primarily to originate from COVID-19; are unable to provide informed consent.

Data collection

<u>Phase 1</u>

Face-to-face semi-structured in-depth interviews with eligible participants will explore their experiences of living with CRD, including functional limitations, and identify potential enablers and barriers to participating in or adhering to PR. These interviews will be conducted in a quiet, private room. Prior to each interview, the reseachers will explain the study to each participant using a detailed participant information sheet (supplementary file 1), written in plain Chichewa (most used language in Malawi) and English, so that each participant can make an informed decision to participate or not. The researchers will read the participant information sheet loudly and verbatim at a moderate pace. This will be followed by a question-and-answer session between the participant and reseachers, respectively, to ensure that all the participant’s questions and concerns about the study are satisfactorily addressed by the reseachers. Then, the participant will be asked to give their

For peer review only - http://bmjopen.bmj.com/site/about/guidelines.xhtml

written informed and autonomous consent to participate or not. Interviews are expected to last for up to one hour and will be audio-recorded with prior consent of each participant. A topic guide (Supplementary file 2) will direct the content of the interview and will include participants' experiences with breathing difficulties, anticipated enabling and limiting factors to undertaking a PR programme, attitudes and views on different elements or components of the programme, and suggestions for making the programme culturally acceptable to them.

Phase 2

As already mentioned, phase 1 participants will be invited to participate in phase 2 of the study. A case report form will collect the following data from phase 2 participants (i.e., through self-reported data and measurements) and their medical files:

- **Demographic variables** including age, sex, ethnic group, highest education level and employment status;
- **Lung health** variables including potential risk factors for lung disease (these will be assessed by adapting IMPALA questionnaires (28) available at https://github.com/jipp3r/IMPALA_QuestionSet, e.g., smoking status (pack years) andbiomass fuel exposure), primary respiratory diagnosis, time since diagnosis in years, number of CRD exacerbations and hospitalisations within the last 12 months, spirometric measurements (pre- and post-bronchodilator FEV_1, FVC, and FEV_1/FVC ratio); comorbidities and treatments;
- **Anthropometric measurements** of height (using a Holtain stadiometer), weight (using calibrated weight scale), and body mass index (BMI);
- **Health status** variables including participants' perceived respiratory disability due to dyspnoea (using the mMRC dyspnoea scale (29)), health-related quality of life (using the COPD Assessment Test (30)), subjective experience of fatigue (using the Checklist Individual Strength fatigue subscale (31)), and psychological wellbeing (using the Hospital Anxiety and Depression Scale (32)) – these tools will be translated and

For peer review only - http://bmjopen.bmj.com/site/about/guidelines.xhtml

For peer review only

administered to participants in a standard manner in Chichewa, with the caveat being that they have not been cross-culturally validated in Malawi;

- **Physical fitness** variables including exercise tolerance (using the incremental shuttle walk test (33)), with before- and after-test measurements of oxygen saturation (SpO_2) and heart rate (using a pulse oximeter), blood pressure if not recently documented in the participant's medical file (using a sphygmomanometer), Borg rating of breathlessness (34), and Borg rate of perceived exertion (34). We will also assess lower extremity muscular strength (using five-repetition sit-to-stand test (35)) and hamstring flexibility (using the chair sit-and-reach test (36)).

Using combination of researcher observation, interaction with the participants, field notes and informal interviews, we will also assess the feasibility of running the PR programme in this study in the following areas: participants' recruitment (i.e., availability of eligible participants and their referral to our PR programme); retention (i.e., number and reasons of participant dropouts); engagement (i.e., participants' enthusiasm with the intervention); and protocol adherence to the exercise regimen at both home and hospital.

Intervention description

Interview data from phase 1 will be used to culturally adapt the PR programme. We initially propose a six-week, twice-weekly, supervised centre-based PR programme, with an additional weekly home-based non-supervised session. The programme will be delivered in a group format and will consist of about an hour of exercise training and another hour of health education. The PR sessions or classes will be run in the physiotherapy department at QECH. Participants will be provided with refreshments (i.e., during rest periods of exercise sessions) and reimbursed for transport at rates mandated by the local research ethics committee. The initial intensity of the exercise regime will be tailored to each participant's exercise capacity (i.e., using incremental shuttle walk test performance results for individual participant, i.e., 50%–75% of their peak speed achieved on the incremental shuttle walk test (37)), and gradually increased according to each patient's improvement over the course of PR (e.g., as

For peer review only - http://bmjopen.bmj.com/site/about/guidelines.xhtml

they are able to do more repetitions of an exercise than they were on the previous session)(38).

At each session, participants will complete a set of exercises designed to improve aerobic fitness, muscle strength, endurance, and flexibility. Established principles of exercise training will be used during exercise prescription (39). Each participant will also be given an information sheet containing illustrations and instructions written in Chichewa for their weekly home-based exercise session. Participants will be requested to keep a standardized exercise and physical activity diary at home, which will have been pilot-tested a priori. The PR programme will not interfere with the participants' routine medical care from the primary care and/or referring teams.

The education component will be delivered to participants and their families or caregivers, which address: patients as active participants in their health care; understanding of the physical and psychological changes that occur with chronic illness, and coping strategies for these (40). Education will be delivered either in group or individual sessions depending on the sensitivity of the topic and insights from phase 1. Table 1 below lists some of the topics that may be covered (41).

Table 1: Education topics

Topics that may be suitable for group sessions	Topics that may require individual consultation
Information about the lungs, lung diseases and respiratory medications	Smoking cessation
Exacerbations: prevention and management including action plans	Inhaler technique
Benefits of physical activity and exercise	Nutrition
Causes and management of breathlessness	Oxygen therapy
Coping with chronic lung disease, management of depression, anxiety, and panic attacks	Instruction in airway clearance techniques
Communication with health professionals	Issues related to travelling with lung disease
Community resources including home care options	Sexual intimacy
	Continence
	Advanced care planning and end-of-life decision-making

For peer review only

Phase 3

Repeat assessments of participants' lung function, anthropometry (weight, height, body mass index), submaximal exercise tolerance, and health status will be conducted. Post-trial interviews with participants will also be conducted using a topic guide (Supplementary file 3), to assess the acceptability of the programme to participants. All participants who either complete or do not complete the six-week PR programme will be invited to participate in these interviews. They will be asked for what went well and what might be improved in the programme. The interviews will be conducted in a private room at QECH (or at participant's home if they are unable to travel to QECH due to illness, etc), and are expected to last for up to one hour and will be audio-recorded with prior consent.

Patient and public involvement

As described in phase 1 and 2 sections, patients will be involved in the design of a culturally appropriate PR programme for this trial using their data collected through in-depth interviews prior to PR trial phase. After the trial, we will recruit a patient and public involvement (PPI) representative (patient expert) from the trial participants' group who can help, in a formal role, with the dissemination of trial findings and PPI in future studies. Currently, the protocol has received both internal and external expert input.

Data management

The CRFs will be printed out so that participants' data collected by these will be stored in physical form. All CRFs with participants' data will be stored securey in a locked cabinet in the Physiotherapy Department at QECH. These will later be anonmisyed and digitally archived on a public online database similar to IMPALA's peer-reviewed "Questionnaires for Lung Health across the Life Course" (https://github.com/jipp3r/IMPALA_QuestionSet)(42), as a harmonized and shared data collection system of demographics, baseline and PR outcomes for patients undergoing PR in Malawi. The management of the system will be supported by the Data Management Support Unit at the Malawi-Liverpool-Wellcome Trust Clinical Research Programme. Likewise, interview data will be anonymised at the time of translation and transcription (i.e., participants' names and dates will be removed). All anonymised transcripts

For peer review only - http://bmjopen.bmj.com/site/about/guidelines.xhtml

will be stored for a maximum of five years on a password-encrypted computer at Malawi-Liverpool-Wellcome Trust Clinical Research Programme, after which they will be permanently deleted.

Quantitative data analysis and presentation

Quantitative data will be imported to IBM SPSS Statistics 27 for descriptive analysis. The average pre-post PR outcome differences or changes in clinical outcomes will be represented as relative and absolute changes. Non-parametric statistical testing (Wilcoxon signed-rank test (43)) will be used to explore differences, with the caveat that this feasibility trial is not designed a priori for statistical power to detect differences or changes in clinical outcomes. In addition, as the trial lacks a control group with which to compare any recorded difference in clinical outcomes, we will be unable to attribute any pre-post PR difference or change in clinical outcomes to PR. However, where appropriate, the average changes in clinical outcomes will be discussed in relation to published minimal clinically important differences (44). Results from quantitative data analysis will be presented in tables and/or graphs, with mean (standard deviation), median (inter-quartile range) or frequency (percentage) used to report summary data.

Qualitative data analysis and presentation

Anonymised interview transcripts will be imported into QSR NVivo software platform for thematic analysis (45). The analysis will be performed collaboratively between researchers to encourage a breadth and depth of analysis from different perspectives and viewpoints. Initially, the researchers will collaborate to develop a preliminary coding framework. Data coding will be performed by the researchers separately, using an iterative inductive approach (45) across all qualitative data. In summary, the researchers will broadly follow the steps outlined by Braun and Clarke (45), including going through a process of familiarization with the data by reading and re-reading transcripts while making reflective notes on the literal content, looking closely at words used by participants, interpreting what the data meant by assigning initial codes or classifications to segments of text, and exploring relationships between these classifications and developing

For peer review only - http://bmjopen.bmj.com/site/about/guidelines.xhtml

core themes. Results from qualitative data analysis will be presented in narrative form based on key themes established during the analysis, supplemented by participants' quotations.

Adverse events

For the purposes of this work, an adverse event is defined as any unfavourable or unintended sign or response associated with participating in the PR programme of this trial. Due to anticipated thorough participant assessment (including risk assessment) and individualised nature of PR in this trial, adverse events are not anticipated. However, if such events do occur, the programme will be stopped immediately for the affected participant so that they can be given appropriate management, which may include referral to the medical team. All adverse events will be registered on the adverse event log and CRF. Serious adverse events will be reported to the Clinical Research Support Unit (CRSU) at Malawi-Liverpool-Wellcome Trust Clinical Research Programme, COMREC, and University of Leicester Research Ethics Committee, for guidance where required.

Ethics and dissemination of the results

Ethical approval for this trial was obtained from University of Malawi College of Medicine Research and Ethics Committee (COMREC), Blantyre, Malawi (protocol number: P.07/19/2752), and University of Leicester Research Ethics Committee, Leicester, United Kingdom (ethics reference: 31574). The results of the trial will be disseminated through oral presentations at local and international scientific conferences or seminars and publication in a peer-reviewed journal. We will also engage the participants who complete the PR trial and the Science Communication Department at Malawi-Liverpool-Wellcome Trust Clinical Research Programme to organise community outreach activities within Blantyre to educate communities about CRDs and PR. We will also broadcast our trial results through national radio station programmes such as the weekly "Thanzi la Onse" (Health of All) programme by Times Radio Malawi. We will formally present our trial results to Blantyre District Health Office and Malawi Ministry of Health.

For peer review only - http://bmjopen.bmj.com/site/about/guidelines.xhtml

Authors' contributions

FMB drafted the manuscript based on the original study protocol conceived and designed by FMB, JR andEC. FMB sent the draft manuscript to JR, EC, TM, RN, MWO, CF, MM, LM, WL, LW, IP, ZY, ZA, MK, FL, HS, AB, RCF, MCS, JM, AM, and SJS for their critical reviews for important intellectual content. FMB revised the draft accordingly. All authors approved the final revised draft to be submitted to *BMJ Open* and are accountable for all aspects of the work.

Funding

This work was supported by the National Institute for Health Research (NIHR) (using the UK's Official Development Assistance (ODA) Funding) and Wellcome [221465/Z/20/Z] under the NIHR-Wellcome Partnership for Global Health Research; the Royal Society of Tropical Medicine and Hygiene (RSTMH) 2019 Small Grants programme (funded by NIHR; award/grant number N/A); the Academy of Medical Sciences Global Challenges Research Fund (GCRF) Networking Grant Scheme (GCRFNGR5\1242); and the NIHR- Global Health Research Group on Respiratory Rehabilitation (Global RECHARGE) (17/63/20) using UK aid from the UK Government to support global health research. The views expressed are those of the authors and not necessarily those of Wellcome, RSTM, the NIHR or the Department of Health and Social Care.

Competing interests

The authors declare no competing interests.

For peer review only

References

1. Meghji J, Mortimer K, Agusti A, Allwood BW, Asher I, Bateman ED, et al. Improving lung health in low-income and middle-income countries: from challenges to solutions. Lancet. 2021;397(10277):928-40.
2. van Kampen SC, Wanner A, Edwards M, Harries AD, Kirenga BJ, Chakaya J, et al. International research and guidelines on post-tuberculosis chronic lung disorders: a systematic scoping review. BMJ Glob Health. 2018;3(4):e000745.
3. Bickton FM, Fombe C, Chisati E, Rylance J. Evidence for pulmonary rehabilitation in chronic respiratory diseases in sub-Saharan Africa: a systematic review. Int J Tuberc Lung Dis. 2020;24(10):991-9.
4. Nightingale R. Non-communicable respiratory disease in Malawi: a systematic review and meta-analysis. Malawi Medical Journal. 2020;32:64-73.
5. Meghji J, Lesosky M, Joekes E, Banda P, Rylance J, Gordon S, et al. Patient outcomes associated with post-tuberculosis lung damage in Malawi: a prospective cohort study. Thorax. 2020;75(3):269.
6. Meghji J, Gregorius S, Madan J, Chitimbe F, Thomson R, Rylance J, et al. The long term effect of pulmonary tuberculosis on income and employment in a low income, urban setting. Thorax. 2021;76(4):387.
7. Montuschi P, Malerba M, Santini G, Miravitlles M. Pharmacological treatment of chronic obstructive pulmonary disease: from evidence-based medicine to phenotyping. Drug Discov Today. 2014;19(12):1928-35.
8. Kibirige D, Sanya RE, Nantanda R, Worodria W, Kirenga B. Availability and affordability of medicines and diagnostic tests recommended for management of asthma and chronic obstructive pulmonary disease in sub-Saharan Africa: a systematic review. Allergy Asthma Clin Immunol. 2019;15:14.
9. Gimigliano F, Negrini S. The World Health Organization "Rehabilitation 2030: a call for action". Eur J Phys Rehabil Med. 2017;53(2):155-68.
10. Cieza A, Causey K, Kamenov K, Hanson SW, Chatterji S, Vos T. Global estimates of the need for rehabilitation based on the Global Burden of Disease study 2019: a systematic analysis for the Global Burden of Disease Study 2019. Lancet. 2021;396(10267):2006-17.
11. Spruit MA, Singh SJ, Garvey C, ZuWallack R, Nici L, Rochester C, et al. An official American Thoracic Society/European Respiratory Society statement: key concepts and advances in pulmonary rehabilitation. Am J Respir Crit Care Med. 2013;188(8):e13-64.
12. Bolton CE, Bevan-Smith EF, Blakey JD, Crowe P, Elkin SL, Garrod R, et al. British Thoracic Society guideline on pulmonary rehabilitation in adults. Thorax. 2013;68 Suppl 2:ii1-30.
13. Majd S, Apps L, Chantrell S, Hudson N, Eglington E, Hargadon B, et al. A Feasibility Study of a Randomized Controlled Trial of Asthma-Tailored Pulmonary Rehabilitation Compared with Usual Care in Adults with Severe Asthma. The Journal of Allergy and Clinical Immunology: In Practice. 2020;8(10):3418-27.

For peer review only - http://bmjopen.bmj.com/site/about/guidelines.xhtml

14. Ando M, Mori A, Esaki H, Shiraki T, Uemura H, Okazawa M, et al. The effect of pulmonary rehabilitation in patients with post-tuberculosis lung disorder. Chest. 2003;123(6):1988-95.
15. Patel S, Cole AD, Nolan CM, Barker RE, Jones SE, Kon S, et al. Pulmonary rehabilitation in bronchiectasis: a propensity-matched study. Eur Respir J. 2019;53(1).
16. Johnston K, Grimmer-Somers K. Pulmonary rehabilitation: overwhelming evidence but lost in translation? Physiother Can. 2010;62(4):368-73.
17. Griffiths TL, Phillips CJ, Davies S, Burr ML, Campbell IA. Cost effectiveness of an outpatient multidisciplinary pulmonary rehabilitation programme. Thorax. 2001;56(10):779-84.
18. Patel S, Palmer MD, Nolan CM, Barker RE, Walsh JA, Wynne SC, et al. Supervised pulmonary rehabilitation using minimal or specialist exercise equipment in COPD: a propensity-matched analysis. Thorax. 2021;76(3):264-71.
19. Habib GMM, Rabinovich R, Divgi K, Ahmed S, Saha SK, Singh S, et al. Systematic review of clinical effectiveness, components, and delivery of pulmonary rehabilitation in low-resource settings. NPJ Prim Care Respir Med. 2020;30(1):52.
20. Singh SJ, Halpin DMG, Salvi S, Kirenga BJ, Mortimer K. Exercise and pulmonary rehabilitation for people with chronic lung disease in LMICs: challenges and opportunities. Lancet Respir Med. 2019;7(12):1002-4.
21. Habib GM, Uzzaman MN, Malik P, Rabinovich R, Uddin A, Alam SR, et al. Engaging with stakeholders in a research programme to promote implementation of pulmonary rehabilitation in Bangladesh: Challenges and opportunities. J Glob Health. 2020;10(2):020384.
22. Sekhon M, Cartwright M, Francis JJ. Acceptability of healthcare interventions: an overview of reviews and development of a theoretical framework. BMC Health Services Research. 2017;17(1):88.
23. Zyl C, Badenhorst M, Hanekom S, Heine M. Unravelling 'low-resource settings': a systematic scoping review with qualitative content analysis. British Medical Journal Global Health. 2021;6:e5190.
24. Chan AW, Tetzlaff JM, Altman DG, Laupacis A, Gøtzsche PC, Krleža-Jerić K, et al. SPIRIT 2013 statement: defining standard protocol items for clinical trials. Ann Intern Med. 2013;158(3):200-7.
25. Clarke H, Voss M. The role of a multidisciplinary student team in the community management of chronic obstructive pulmonary disease. Prim Health Care Res Dev. 2016;17(4):415-20.
26. Jones RC, Copper S, Riley O, Dobbs F. A pilot study of pulmonary rehabilitation in primary care. Br J Gen Pract. 2002;52(480):567-8.
27. NICE. Chronic obstructive pulmonary disease in over 16s: diagnosis and management. 2018.
28. Saleh S, van Zyl-Smit R, Allwood B, Lawin H, Mbatchou Ngahane BH, Ayakaka I, et al. Questionnaires for Lung Health in Africa across the Life Course. Int J Env Res Public Health. 2018;15(8):1615.

For peer review only - http://bmjopen.bmj.com/site/about/guidelines.xhtml

29. Munari AB, Gulart AA, dos Santos K, Venâncio RS, Karloh M, Mayer AF. Modified Medical Research Council Dyspnea Scale in GOLD Classification Better Reflects Physical Activities of Daily Living. Respiratory Care. 2018;63(1):77.
30. Jones PW, Harding G, Berry P, Wiklund I, Chen WH, Kline Leidy N. Development and first validation of the COPD Assessment Test. Eur Respir J. 2009;34(3):648-54.
31. Vercoulen JH, Swanink CM, Fennis JF, Galama JM, van der Meer JW, Bleijenberg G. Dimensional assessment of chronic fatigue syndrome. J Psychosom Res. 1994;38(5):383-92.
32. Zigmond AS, Snaith RP. The hospital anxiety and depression scale. Acta Psychiatr Scand. 1983;67(6):361-70.
33. Singh SJ, Morgan MD, Scott S, Walters D, Hardman AE. Development of a shuttle walking test of disability in patients with chronic airways obstruction. Thorax. 1992;47(12):1019-24.
34. Mahler DA, Horowitz MB. Perception of breathlessness during exercise in patients with respiratory disease. Med Sci Sports Exerc. 1994;26(9):1078-81.
35. Jones SE, Kon SSC, Canavan JL, Patel MS, Clark AL, Nolan CM, et al. The five-repetition sit-to-stand test as a functional outcome measure in COPD. Thorax. 2013;68(11):1015.
36. Jones CJ, Rikli RE, Max J, Noffal G. The reliability and validity of a chair sit-and-reach test as a measure of hamstring flexibility in older adults. Res Q Exerc Sport. 1998;69(4):338-43.
37. Zainuldin R, Mackey MG, Alison JA. Prescription of walking exercise intensity from the incremental shuttle walk test in people with chronic obstructive pulmonary disease. Am J Phys Med Rehabil. 2012;91(7):592-600.
38. Armstrong M, Vogiatzis I. Personalized exercise training in chronic lung diseases. Respirology. 2019;24(9):854-62.
39. Garvey C, Bayles MP, Hamm LF, Hill K, Holland A, Limberg TM, et al. Pulmonary Rehabilitation Exercise Prescription in Chronic Obstructive Pulmonary Disease: Review of Selected Guidelines: AN OFFICIAL STATEMENT FROM THE AMERICAN ASSOCIATION OF CARDIOVASCULAR AND PULMONARY REHABILITATION. J Cardiopulm Rehabil Prev. 2016;36(2):75-83.
40. Australia" LF. Pulmonary rehabilitation toolkit – importance of education. 2016
41. Jenkins S, Hill K, Cecins NM. State of the art: how to set up a pulmonary rehabilitation program. Respirology. 2010;15(8):1157-73.
42. Saleh S, van Zyl-Smit R, Allwood B, Lawin H, Mbatchou Ngahane BH, Ayakaka I, et al. Questionnaires for Lung Health in Africa across the Life Course. Int J Environ Res Public Health. 2018;15(8).
43. King AP, Eckersley RJ. Chapter 6 - Inferential Statistics III: Nonparametric Hypothesis Testing. In: King AP, Eckersley RJ, editors. Statistics for Biomedical Engineers and Scientists: Academic Press; 2019. p. 119-45.
44. McGlothlin AE, Lewis RJ. Minimal Clinically Important Difference: Defining What Really Matters to Patients. JAMA. 2014;312(13):1342-3.
45. Braun V, Clarke V. Using thematic analysis in psychology. Qualitative Research in Psychology. 2006;3(2):77-101.

For peer review only

Supplementary file 1: Participant Information Sheet (English version)

1. **Study title**

Feasibility and acceptability of pulmonary rehabilitation for adults with functionally limiting chronic respiratory disease in Malawi: A mixed-methods single-arm interventional prospective cohort study

2. **Invitation**

I would like to invite you to take part in a research study. Before you decide you need to understand why the research is being done and what it would involve for you. Please take time to read the following information carefully. Ask questions if anything you read is not clear or would like more information. Take time to decide whether or not to take part.

3. **Purpose of study**

The study aims to (1) establish the acceptability and feasibility of an intervention in the management of adults with functionally limiting chronic respiratory diseases (CRDs) in Malawi, called pulmonary rehabilitation (PR), and (2) to design a locally appropriate PR program for adults with functionally limiting CRDs in Malawi. A similar study has recently been conducted in Uganda.

4. **Why have I been invited?**

You have been invited to participate in this study because you have a chronic respiratory disease.

5. **Do I have to take part?**

Participation in the study is entirely voluntary. It is up to you to decide. We will describe the study and go through the information sheet, which we will give to you. We will then ask you to sign a consent form to show you agreed to participate. You are free to withdraw at any time, without giving a reason and this will not affect the standard of care you receive for your disease.

6. **What will happen to me if I take part?**

You will be invited to participate and complete a pulmonary rehabilitation (PR) programme run at Queen Elizabeth Central Hospital (QECH) by a team of healthcare professionals including physiotherapists, nurses and doctors. Before the programme begins, you will be assessed for your eligibility to participate in the study and to establish your baseline characteristics. The programme will run for 6 weeks, 2 days of a supervised session each week at QECH and 1 day of unsupervised session as your home, 2 hours each session. The programme will involve exercises such as walking, education such as knowledge about your disease, and behaviour change such as smoking cessation. The exercises will be tailored and progressed according to each participant's abilities. Other 9 participants of a similar condition like yours will also participate in the study together with you. After the 6 weeks of the programme, you will be assessed again for any changes that might have happened due to the programme. We may also assess you during the programme. The assessments may involve, among others, your lung function, your exercise capacity/tolerance, your quality of life, and vital signs such as blood pressure and pulse rate. There will be social interaction with other participants during the programme. There will also be rest periods and provision of light food and soft drinks as refreshments. We will try to

For peer review only - http://bmjopen.bmj.com/site/about/guidelines.xhtml

make the programme as much interesting and enjoyable as possible to everyone. At times, we will collect information from you about the programme through audio-recorded interviews and group discussions. All data collected will be managed with confidentiality by the study team.

7. **Will there be any risks involved in the study?**

PR is safe except under certain circumstances, such as if you have uncontrolled heart disease or exacerbations of your respiratory diseases. The assessment we will conduct prior to your participation in the program will help us establish the programme's safety to you. In the case where exacerbations happen to you during the programme, we will immediately stop the programme and refer you to the medical personnel for immediate treatment. Since the programme will happen within the premises if the hospital, such referral process will be easier for you. The programme will not stop you from continuing your normal standard of care including taking your medications. Otherwise, with all safety measures in place, we don't expect any risks during the study.

8. **Will there be any benefits involved in being in the study?**

We cannot promise the study will help you but we know that PR has well-established benefits. We, therefore, hope that it will lead to better outcomes of your disease and health.

9. **What will happen to the findings of the study?**

We will record the information we collect from you using a computer. This information will be transferred to a computer database with your identifiers removed, so that you cannot be identified from this information. The information will be stored securely for 5-years.

The results of the study will be shared at research meetings (in Malawi and overseas) and will be published in medical journals, so that other healthcare professionals and patients can benefit from our findings. You will not be identified in such avenues too.

10. **Who is organizing the study?**

The principal investigator of the study is Mr. Fanuel Bickton, a trained physiotherapist and research intern working at the Malawi-Liverpool-Wellcome Trust Clinical Research Programme (MLW). He and the study are being supervised by Dr. Jamie Rylance from MLW and Mr. Enock Chisati from the University of Malawi.

11. **Expenses/payments**

You will be compensated for your travel expenses (MWK1500.00 per study visit) to participate in the study.

12. **Source of funding for the study**

The study is funded by the Malawi-Liverpool-Wellcome Trust Clinical Research Programme through the Lung Health research group.

13. **What happens if I change my mind?**

If you agree to join the study you can change your mind and withdraw your consent at any time. If you have any questions about this study, please contact any of the following individuals:

For peer review only - http://bmjopen.bmj.com/site/about/guidelines.xhtml

Principle investigator:

Mr. Fanuel Bickton:
Cell: +265 982 55 23 53
E-mail: fmbickton@stud.medcol.mw

Supervisors:

1. Dr. Jamie Rylance:
   Cell: +265 (0) 996 80 46 11
   E-mail: jamie.rylance@lstmed.ac.uk

2. Mr. Enock Chisati:
   Cell: +265 (0) 888 16 82 84
   E-mail: echisati@medcol.mw / echisati@gmail.com

The local ethics committee (COMREC) has reviewed and approved this study – any problems that cannot be addressed by the study team, should be directed to:

COMREC Secretariat, College of Medicine, P/Bag 360, Chichiri, Blantyre 3, Malawi; Tel: +265 1871911 ext. 334; E-mail: comrec@medcol.mw

You will be given a copy of this sheet to keep, together with a copy of your consent form.

For peer review only

Supplementary file 2: Topic Guide for Pre-PR In-Depth Interviews (English version)

The guide may be adapted during the research in response to emerging issues.
Overall structure of the interview:

i. Consent of the participant to take part in this trial
ii. **Recorder started**
iii. Self-introduction by the researcher
iv. Explanation to the participant that they should treat the information they hear as confidential
v. Detailed explanation of what PR is and what it involves (see definition and explanation below)
vi. Structured interview around participant's barriers and enablers for participating and completing a PR programme
vii. **Recorder stopped**

PR summary (to use for quick reference for key elements of PR for either interviewer or participant where necessary):

Pulmonary rehabilitation is the use of exercise, education, and behaviour change to improve health and quality of life in people with chronic respiratory diseases such as COPD, asthma and p-TBLD. The exercise component may include walking, the education component may include knowledge about nutrition and patient's disease and how patients can manage the symptoms themselves, and behaviour change may include adopting and adhering to health-enhancing behaviours such as smoking cessation. PR begins and ends with a thorough assessment of the patient. PR is recommended in international guidelines for the management of chronic respiratory diseases; it has been found to improve the patient's quality of life by, among others: reducing shortness of breath, increasing exercise tolerance, promoting a sense of well-being, and decreasing the number of hospitalizations. A typical PR programme runs for at least six weeks and involves weekly two supervised sessions at the hospital and one unsupervised session at the patient's home. Each session takes about 2 hours. Participants interact with each other during the sessions. There are also rest periods and refreshments in form of light food and soft drinks are provided during these periods. We try to make the sessions as much interesting and enjoyable as possible to everyone.

1. **Participant's experience with breathing difficulties**
   1.1 How can you describe your breathing problem?
   1.2 How has your life changed because of breathing difficulties? What is it in your daily life worries you about your breathing?
   1.3 What do you do to relieve your breathing problem?

2. **Participant's views on enabling and limiting factors to undertaking a PR program:** 2.1 What factors do you think would enable you attend and complete the programme?
   2.2 What factors do you think would prevent you from attending and completing the programme?
3. **Participant's attitudes and views on different elements/components of PR:**
   3.1 The programme will be taking place at the hospital's gym, either in the morning or afternoon of the selected 2 days. What can you say and suggest about the location, days, and timing of the programme?

3.2 PR involves group work. What do you think about this?

3.3 One feature of pulmonary rehabilitation is a regular, tailored exercise session. What do you think about this?

3.4 Another feature of the programme is education which may include the importance of smoking cessation along with support to do so. What can you say about this?

3.5 Participating in a PR programme would mean you travelling from your home to a PR centre. What do you say about this?

2.9 A participant group attending a PR program may be a mixture of men and women. How do you feel about that?

2.10 Professionals delivering a PR program can be male or female. What do you say about that?

2.11 Due to the nature of the PR program, you may have to do it in public sometimes. How do you feel about that?

4. Conclusion:

4.1 If you accepted to participate in a PR program, which aspects of your health or life would you most like to see improvement in?

4.2 What are your hopes and doubts about the PR program?

4.3 How can we make the programme successful or attainable for you?

4.4 Do you have additional comments?

Supplementary file 3: Topic guide for post-PR in-depth Interviews (English version)

The guide may be adapted during the research in response to emerging issues.

Overall structure of the interview:

i. Consent of the participant to take part in this trial
ii. **Recorder started**
iii. Self-introduction by the researcher
iv. Explanation to the participant that they should treat the information they hear as confidential
v. Detailed introduction of the interview (see below)
vi. Structured interview around the impact of PR on patient's disease and/or quality of life, barriers, and enablers for participating and completing the trial's PR programme, the willingness/motivation to undertake the programme again in the future, the willingness to continue the programme at home, and the willingness to recommend the programme to other patients with similar condition
vii. **Recorder stopped**

Introduction:

Thank you for participating and completing a PR programme in the trial that has just ended. Now, this part of the trial aims to establish your experiences with the programme including its impact on your health or quality of life, your views on what went well and what went wrong with the programme, your recommendations on how to improve the programme, and your motivation/willingness to undertake the programme again in the future and to continue it at your home. Please, note that there aren't any right or wrong answers. I am just trying to gather your opinions and experiences. I am asking the same questions to your friends with whom you undertook the programme (if IDIs are used) and the information you share with me is confidential.

1. Impact of the PR program on participants

1.1 How are you feeling now?

1.2 How does you disease affect you since you have taken part in the rehabilitation programme?

1.3 What has changed since last time I spoke to you?

1.4 Which aspects of your activities have changed?

1.5 How do you now feel about taking part in activities such as going out/walking, etc.?

1.6 How do you feel about your breathlessness now?

2. Recommendations

2.1 What do you think went well and what did not go well with the programme?

2.2 What recommendations can you suggest improving the program?

2.3 In the future, for you to attend and complete a PR programme, what circumstances would have to change for you?

3. Maintenance of PR program

3.1 Would you consider continuing the programme at your home?

4. **Conclusion**

4.1 Are there any other issues you would like to raise?

CONSORT 2010 checklist of information to include when reporting a pilot or feasibility trial*

Section/Topic	Item No	Checklist item	Reported on page No
**Title and abstract**			
	1a	Identification as a pilot or feasibility randomised trial in the title	1
	1b	Structured summary of pilot trial design, methods, results, and conclusions (for specific guidance see CONSORT abstract extension for pilot trials)	2-3
**Introduction**			
Background and objectives	2a	Scientific background and explanation of rationale for future definitive trial, and reasons for randomised pilot trial	4-6
	2b	Specific objectives or research questions for pilot trial	5-6
**Methods**			
Trial design	3a	Description of pilot trial design (such as parallel, factorial) including allocation ratio	7-8
	3b	Important changes to methods after pilot trial commencement (such as eligibility criteria), with reasons	7-13
Participants	4a	Eligibility criteria for participants	8
	4b	Settings and locations where the data were collected	7
	4c	How participants were identified and consented	7-9
Interventions	5	The interventions for each group with sufficient details to allow replication, including how and when they were actually administered	10-12
Outcomes	6a	Completely defined prespecified assessments or measurements to address each pilot trial objective specified in 2b, including how and when they were assessed	9-10
	6b	Any changes to pilot trial assessments or measurements after the pilot trial commenced, with reasons	9-10
	6c	If applicable, prespecified criteria used to judge whether, or how, to proceed with future definitive trial	N/A
Sample size	7a	Rationale for numbers in the pilot trial	8
	7b	When applicable, explanation of any interim analyses and stopping guidelines	N/A
Randomisation:			
Sequence generation	8a	Method used to generate the random allocation sequence	N/A
	8b	Type of randomisation(s); details of any restriction (such as blocking and block size)	N/A
Allocation concealment mechanism	9	Mechanism used to implement the random allocation sequence (such as sequentially numbered containers), describing any steps taken to conceal the sequence until interventions were assigned	N/A

For peer review only

Implementation	10	Who generated the random allocation sequence, who enrolled participants, and who assigned participants to interventions	N/A
Blinding	11a	If done, who was blinded after assignment to interventions (for example, participants, care providers, those assessing outcomes) and how	N/A
	11b	If relevant, description of the similarity of interventions	N/A
Statistical methods	12	Methods used to address each pilot trial objective whether qualitative or quantitative	13-14
**Results**			
Participant flow (a diagram is strongly recommended)	13a	For each group, the numbers of participants who were approached and/or assessed for eligibility, randomly assigned, received intended treatment, and were assessed for each objective	N/A
	13b	For each group, losses and exclusions after randomisation, together with reasons	N/A
Recruitment	14a	Dates defining the periods of recruitment and follow-up	7
	14b	Why the pilot trial ended or was stopped	N/A
Baseline data	15	A table showing baseline demographic and clinical characteristics for each group	N/A
Numbers analysed	16	For each objective, number of participants (denominator) included in each analysis. If relevant, these numbers should be by randomised group	N/A
Outcomes and estimation	17	For each objective, results including expressions of uncertainty (such as 95% confidence interval) for any estimates. If relevant, these results should be by randomised group	N/A
Ancillary analyses	18	Results of any other analyses performed that could be used to inform the future definitive trial	N/A
Harms	19	All important harms or unintended effects in each group (for specific guidance see CONSORT for harms)	N/A
	19a	If relevant, other important unintended consequences	N/A
**Discussion**			
Limitations	20	Pilot trial limitations, addressing sources of potential bias and remaining uncertainty about feasibility	3, 13-14
Generalisability	21	Generalisability (applicability) of pilot trial methods and findings to future definitive trial and other studies	3 and 6
Interpretation	22	Interpretation consistent with pilot trial objectives and findings, balancing potential benefits and harms, and considering other relevant evidence	N/A
	22a	Implications for progression from pilot to future definitive trial, including any proposed amendments	N/A
**Other information**			
Registration	23	Registration number for pilot trial and name of trial registry	3 and 7
Protocol	24	Where the pilot trial protocol can be accessed, if available	N/A
Funding	25	Sources of funding and other support (such as supply of drugs), role of funders	16
	26	Ethical approval or approval by research review committee, confirmed with reference number	3 and 15

For peer review only

Citation: Eldridge SM, Chan CL, Campbell MJ, Bond CM, Hopewell S, Thabane L, et al. CONSORT 2010 statement: extension to randomised pilot and feasibility trials. BMJ. 2016;355.
*We strongly recommend reading this statement in conjunction with the CONSORT 2010, extension to randomised pilot and feasibility trials, Explanation and Elaboration for important clarifications on all the items. If relevant, we also recommend reading CONSORT extensions for cluster randomised trials, non-inferiority and equivalence trials, non-pharmacological treatments, herbal interventions, and pragmatic trials. Additional extensions are forthcoming: for those and for up to date references relevant to this checklist, see www.consort-statement.org.

For peer review only
